# Strategies for Wheat Stripe Rust Pathogenicity Identified by Transcriptome Sequencing

**DOI:** 10.1371/journal.pone.0067150

**Published:** 2013-06-26

**Authors:** Diana P. Garnica, Narayana M. Upadhyaya, Peter N. Dodds, John P. Rathjen

**Affiliations:** 1 Research School of Biology, Australian National University, Canberra, Australian Capital Territory, Australia; 2 Division of Plant Industry, Commonwealth Scientific and Industrial Research Organisation (CSIRO), Canberra, Australian Capital Territory, Australia; University of Texas, United States of America

## Abstract

Stripe rust caused by the fungus *Puccinia striiformis* f.sp. *tritici* (*Pst*) is a major constraint to wheat production worldwide. The molecular events that underlie *Pst* pathogenicity are largely unknown. Like all rusts, *Pst* creates a specialized cellular structure within host cells called the haustorium to obtain nutrients from wheat, and to secrete pathogenicity factors called effector proteins. We purified *Pst* haustoria and used next-generation sequencing platforms to assemble the haustorial transcriptome as well as the transcriptome of germinated spores. 12,282 transcripts were assembled from 454-pyrosequencing data and used as reference for digital gene expression analysis to compare the germinated uredinospores and haustoria transcriptomes based on Illumina RNAseq data. More than 400 genes encoding secreted proteins which constitute candidate effectors were identified from the haustorial transcriptome, with two thirds of these up-regulated in this tissue compared to germinated spores. RT-PCR analysis confirmed the expression patterns of 94 effector candidates. The analysis also revealed that spores rely mainly on stored energy reserves for growth and development, while haustoria take up host nutrients for massive energy production for biosynthetic pathways and the ultimate production of spores. Together, these studies substantially increase our knowledge of potential *Pst* effectors and provide new insights into the pathogenic strategies of this important organism.

## Introduction

Stripe rust is an important agricultural disease that constitutes a major challenge to wheat production worldwide. The causal agent is the fungus *Puccinia striiformis* f.sp. *tritici (Pst).* In Australia, wheat stripe rust causes severe losses and is controlled mainly by fungicides, with about AUD$127 million expended per annum on chemical strategies [1], [2]. In a worldwide context, the disease has become increasingly important in the key wheat-consuming areas of North Africa, west and central Asia [3], and China [4], and in the U.S [5] where epidemics have caused huge yield losses since 2000. The economic importance of rusts derives from three main factors; the large extent to which they can reduce grain yield, their ability to spread rapidly and reach epidemic proportions under favorable conditions, and their rapid mutation to overcome host resistance genes [6]. Thus, understanding the molecular strategies used by *Pst* to cause disease is crucial for developing more effective control methods.

Rust fungi have the most complicated life cycles of all fungi [7]. Recently, the discovery of the alternate host of stripe rust has allowed classification of this pathogen as macrocyclic because it produces five types of spores during a complete life cycle, and heteroecious, because it requires two hosts for completion of the cycle [8]. Uredinospores produced during the asexual cycle are infectious on wheat, and multiple cycles of reinfection are possible during the growing season. Rust fungi, including *Pst*, are obligate biotrophic pathogens that require living tissue to develop and reproduce. The biotrophic interaction may be sustained over many weeks, with important implications for the pathogenic lifestyle. For example, such prolonged interaction is usually associated with changes in photosynthate translocation patterns within the plant [9]. The infection site becomes a nutrient sink allowing the parasite to exploit the plant’s resources [10]. Plants constantly monitor their local environment and induce defensive responses against invasive microbes, including hypersensitive cell death that is antithetical to biotrophy. Thus the need to survive the inhospitable inner leaf environment while mining the available nutrients may encapsulate the major forces impacting the evolution of biotrophy [11].

As an obligate parasite, *Pst* has adapted to source nutrients from living mesophyll wheat cells through a sophisticated cellular structure termed the haustorium [12]. This arises as a differentiation of the infectious hyphae and invades the host cell, however it is not located within plant cell *per se* but is separated from the host cytoplasm by an extrahaustorial membrane which is contiguous with the plant cell plasma membrane. The haustorium is thought to be the primary organ for nutrient transfer from the host cell to the fungal vegetative body [13], [14]. The space between the extrahaustorial membrane and fungal haustorial wall, called the extrahaustorial matrix, is enriched in carbohydrates with unknown function in parasitism [12]. Haustoria are not only feeding structures; they induce structural changes in the host cell including cytoskeletal rearrangements, nuclear migration and chromatin condensation [15], and there is evidence that they influence host cell metabolism [16], [17]. Furthermore, they deliver essential virulence molecules called ‘effectors’ into the extrahaustorial matrix, several of which are subsequently translocated into host cells [18], [19].

The effectors secreted by plant pathogens are believed to be essential to the parasitic lifestyle. Very little is known about the activities of plant fungal effectors, but broadly they are thought to manipulate the physiological and immune responses of host cells during infection [20]. Conversely, in certain cases the host has evolved to recognize effectors through the action of specific intracellular immune receptors. In this case, the recognized effector serves as a signal for the plant to induce defenses to block pathogen growth [21]. Historically, such effectors are termed avirulence (Avr) proteins.

The haustorium is a site of concerted host-pathogen interaction, and describing its functions is essential to understanding biotrophy. The presence of sugar transporters [14] and putative amino acid transporters [13], [22] within the structure implies an important role in fungal nutrient uptake. However, some other basic questions regarding the function of the haustorium have yet to be addressed. These include the identification of mechanisms for bidirectional transport (importation of nutrients and export of effectors and other molecules), the pathogen and host proteins specifically located at the haustorium–host cell interface, and the regulatory genes which specify haustorial identity.

The availability of genomic data for biotrophic organisms has increased dramatically in the last decade providing important insights into the infection strategies of these pathogens. The genome sequences for the plant pathogens *Puccinia graminis* f.sp *tritici* (*Pgt*), *Melampsora larici-populina* (*Mlp*) [23] and *Blumeria graminis* [24] share some evolutionary features of adaption to the extreme parasitic lifestyle, such as the loss of nitrate and sulfate assimilation pathways. Expressed sequence tag (EST) libraries have been generated for *Pst* [25], [26], [27] and other *Puccinia* species [28], [29], [30], which have been useful for stage-specific expression analysis and gene prediction in genomes. However, the resolution power of these studies for completeness and comparison of transcript abundance between cell types is limited. Here, we have taken advantage of next generation-sequencing (NGS) technologies and haustoria purification methods [31] to broadly analyze gene expression in the germinated uredinospores (henceforth called “germinated spores”)and haustorial stages of *Pst.* The results derived from 454 and Illumina-based transcriptome sequencing allowed the prediction of haustorial secreted proteins (HSP), a set of genes enriched in effector candidates found to be specifically- or highly-expressed in haustoria. Additionally, the comparison of the haustorial and germinated spores transcriptomes revealed fundamental metabolic differences between the two pathogenic stages.

## Results and Discussion

### 454-pyrosequencing and Assembly

Transcriptomes corresponding to purified stripe rust haustoria and germinated spores were sequenced by single-read pyrosequencing on a 454 GS-FLX titanium platform (454). This produced 729,036 and 457,071 reads averaging 413 bp and 420 bp in length for haustoria and germinated spores, respectively. To remove contaminating wheat sequences from the haustoria data, the Pst-130 draft genome [32], a draft genome of a local *Pst* isolate (Pst-104E137A-) (Jackson, Garnica, Foret, Rathjen and Studholme, unpublished data) and the germinated spore transcriptome generated in this study (Text S1 and Data S1) were used as references to extract fungal sequences. Since these references do not represent the complete *Pst* genome, the remaining unmapped reads were assembled *de novo* and the resulting contigs screened by BLAST search against the NCBI nucleotide and protein databases. The BLAST result was curated manually and contigs showing hits to plant genes were removed, while the remaining contigs were retained as novel transcripts not included in the draft genome assemblies. The filtered haustorial reads were then assembled *de novo* using CLC genomics (CLC Bio v3.9) resulting in 12,846 contigs representing the haustorial transcriptome. Assembled contigs ranged between 200 and 6,854 bp, with an average length of 704 bp (Data S2 and Figures S1, S2 and S3).

### Putative Effector Candidate Genes Expressed in Haustoria

The haustoria contigs were analyzed using CLC genomics to identify all possible ORFs, which were further analysed with SignalP 3.0 [33] to predict secretion signal peptides (SP). We found 1,299 SP-encoding genes which were then filtered to include only those unique to a contig, or alternatively the largest SP-ORF within a contig. Proteins containing transmembrane domains (predicted using TMHMM 2.0 [34]) or mitochondrial targeting signals (predicted using TargetP [35]) were excluded. This left 437 haustorial secreted proteins (HSPs, Data S3). The predicted mature peptide sequences (minus the SP) were searched against the NCBI non-redundant protein database using BLASTx, and were additionally analysed with BLAST2GO (B2G) [36] for the presence of conserved InterProScan protein signatures. About 60% of these genes had no similarity to known genes at the level of e-val>10^−25^. One hundred and five genes were similar to hypothetical proteins in *Pgt*, and four were similar to previously identified *Pst* secreted proteins [37]. Forty HSPs could be partially annotated with B2G, fifteen of which were classified as glycoside hydrolases from different families, especially family 18 (chitinase activity) and families related to plant cell wall degradation. Four HSPs were annotated as putative polysaccharide deacetylases (chitin deacetylases), and the rest were annotated as putative proteins participating in diverse cellular processes such as protein folding, proteolysis, oxidation-reduction, and regulation of transcription (Table S2, HSPs). A further 35 HSPs contained conserved protein domains such as zinc finger domain, copper/zinc binding domain, cupredoxin domain, barwin-like endoglucanase domain, NUDIX hydrolase domain, thaumatin and others. Most of these roles or domains have been identifed in other rust predicted secretomes [23], [38], suggesting conserved roles in *Pucciniales*. Interestingly, Bhadauria et. al [39] recently reported a novel effector gene from *Colletotrichum truncatum* (*CtNUDIX*) that is exclusively expressed during the late biotrophic phase and elicits the hypersensitive response in tobacco leaves, suggesting that this effector could be important for the transition from biotrophy to necrotrophy, or alternatively is recognised by the tobacco immune system. A secreted protein from Pst containing a NUDIX domain could potentially regulate redox homeostasis. Nevertheless, as observed previously for rust fungi [38], the absence of recognisable protein domains in candidate effector proteins is a common occurrence.

Genome and transcriptome sequencing of *Blumeria graminis* revealed that the most highly expressed candidate effector proteins contained a Y/F/WxC motif 1–30 aa after the predicted SP [24]. We identified 1 to 4 copies of this sequence motif in 124 of the 437 *Pst* HSPs, but the motif occurred within the first 30 aa for only 43 of these, which is similar to that number expected by chance (∼32). Thus similar to *Pgt* and *Mlp* [23], this motif does not seem to define a major class of effectors in *Pst*. Further attempts to detect novel motifs using the MEME analysis module within CLC failed to identify other conserved sequences.

Extracellular proteins frequently contain elevated numbers of cysteine residues, which can participate in di-sulfide bonding to provide stability to the folded proteins in the protease-rich extracellular apoplastic space. Many known and predicted effector proteins from filamentous pathogens are small cysteine-rich proteins [17], [38], [40]. Analysis of the cysteine content of the predicted 437 HSP proteins revealed that 246 contained fewer than four, and 191 contained from 4–28 cysteines in the mature protein.

### Validation of Haustorial Expression of Effector Candidate Genes

**Figure 1:**
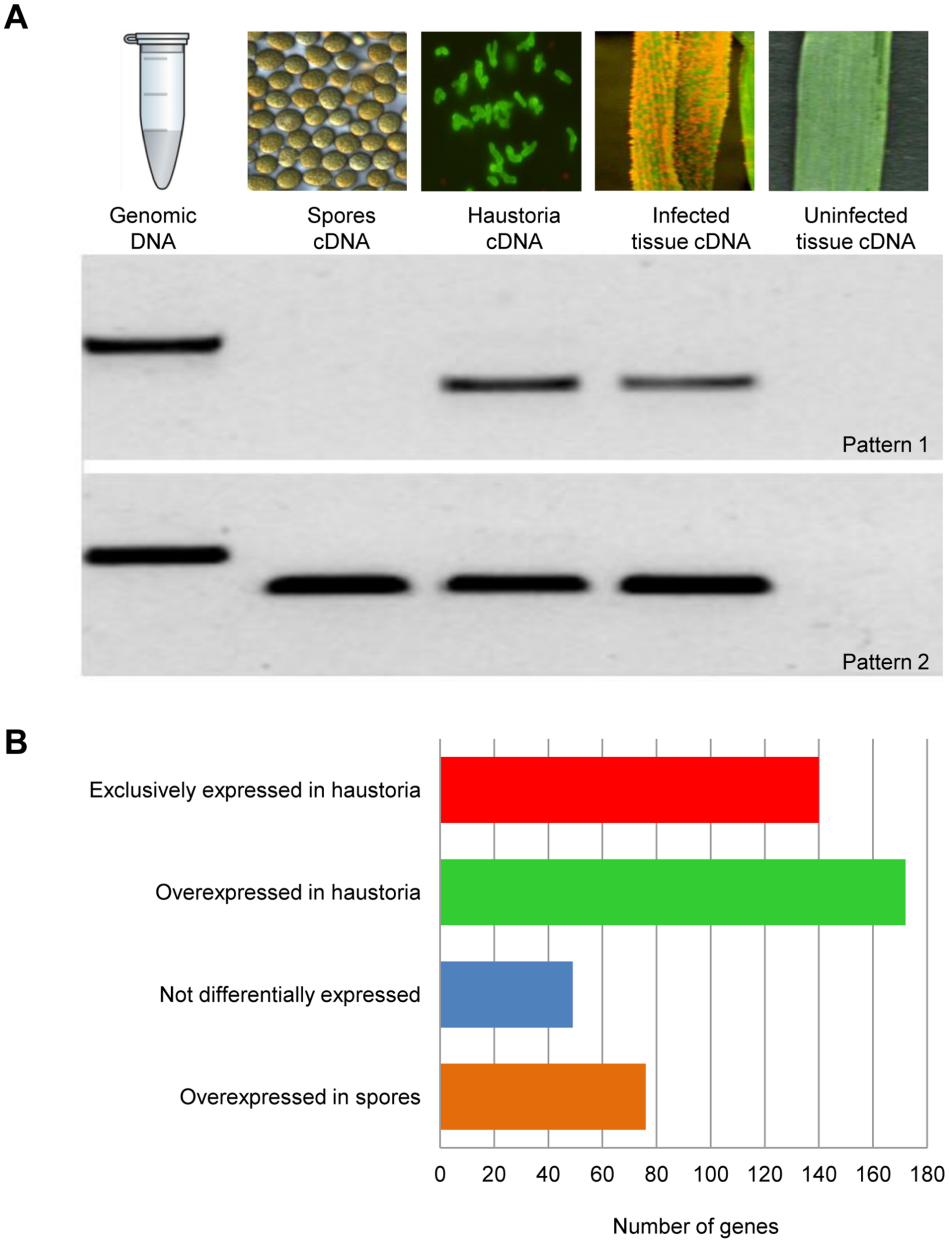
Effector expression patterns. **A.** Ninety four HSPs were selected arbitrarily and their expression patterns were analysed by RT-PCR. Primers were designed to amplify the full length gene sequence minus the signal peptide-encoding region. *Pst* 104E137A- genomic DNA, and cDNAs from germinated spores, isolated haustoria, infected wheat leaves, and uninfected wheat tissue were used as templates. Two expression patterns were obtained: 1. Expression only during the biotrophic phase (Pstv_3161-1), and 2. Expression during early and late stages of development (Pstv_7541-1). None of the tested effector candidates were amplified from uninfected wheat leaves. **B.** Number of HSPs showing digital expression patterns as shown. Overexpression was evaluated using Baggerley’s test [42], genes with a FDR corrected p-value less than 0.05, fold change >2 and a difference of at least 20 were considered to be significant.

To validate the *in silico* predictions (ie to test that the genes are real, are expressed, of the expected size, and of *Pst* origin), 94 effector candidates were randomly chosen from the 437 HSP genes and tested for expression *in vivo* by non-quantitative reverse transcriptase-PCR (RT-PCR)(Figure 1A). Primers were designed to amplify the full ORF excluding the SP-encoding sequence (Table S8, RT-PCR primers). RNA samples were prepared from germinated spores, isolated haustoria, wheat tissue nine days after infection (dai, maximum number of haustoria before sporulation), and uninfected wheat tissue, and subjected to RT-PCR using effector-specific primers. Primer pairs for *Pst* β-tubulin or wheat specific genes served as a positive controls. Seventy one showed expression in isolated haustoria and infected wheat tissue, but no detectable expression in germinated spores, 22 showed expression in all tissues where the fungus was present, and one failed to amplify. Ninety one of the tested genes were confirmed to be of fungal origin in this assay as they were amplified from genomic DNA and/or spore cDNA. The remaining three genes were also confirmed to be fungal origin as they were identified in our draft genome of this isolate (*Pst* 104E137A-) (Jackson et. al. unpublished data) but were not amplified from genomic DNA because one or both primers span intronic regions. Cloning and Sanger sequencing of 25 candidate effectors confirmed that they corresponded to the sequence predicted from our 454 assemblies.

### Digital Expression Analysis of *Pst* Transcriptomes

**Figure 2:**
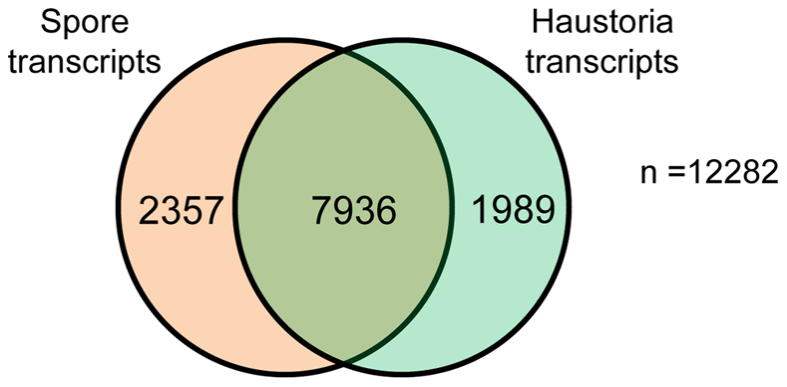
Differential gene expression in *Pst* germinated spores and haustoria. Venn diagram of reference transcripts set, showing the number of transcripts that did not show differential expression between the two tissues, and those that had *statistically significant* changes in *expression* between germinated spores and haustoria.

To compare gene expression between haustoria and germinated spores, we used Illumina RNAseq analysis to obtain statistically robust quantitative expression data. We first assembled a transcriptome reference gene set by *de novo* assembly of the combined germinated spores and haustorial 454 reads, giving a set of 12,282 transcripts (Table S1, Transcripts reference set and Data S4). Three RNA samples comprising independent biological replicates were prepared for each tissue and sequenced by Illumina RNAseq analysis, giving a total of 500 million reads (Table S7). The *Pst* transcripts reference set was used as a template for digital differential expression analysis using the RNAseq tools from CLC genomics. Raw Illumina reads from each tissue were independently mapped against the reference set, and read counts were adjusted to reads per kilobase per million mapped reads (RPKM, [41]), to facilitate the comparison of transcript levels assigned to each gene between samples. RPKMs were statistically assessed using Baggerley’s test [42] (FDR corrected p-value less than 0.05) for each gene to determine differential expression between haustoria and germinated spores. A total of 4,346 transcripts were differentially expressed, revealing a clear difference between the transcriptional programs of germinated spores and haustoria (Table S1 and Figure 2).

Digital expression analysis based on Illumina sequencing also allowed us to quantify the level of expression of the 437 previously predicted HSPs (Table S2, HSPs). All tested genes showed a very close correlation between the expression pattern detected with RT-PCR, and that predicted *in silico* (Table S2, HSPs). Remarkably more than 85% of the HSPs were differentially expressed; 295 overexpressed in haustoria and 76 overexpressed in germinated spores. Strikingly, 40% of the HSPs overexpressed in haustoria showed no expression in germinated spores and 50% were ten or more times expressed in haustoria than in germinated spores, consistent with the idea that effectors play important roles during the biotrophic host-pathogen interaction (Figure 1B).

### Functional Classification of Transcriptome Sequences

**Figure 3:**
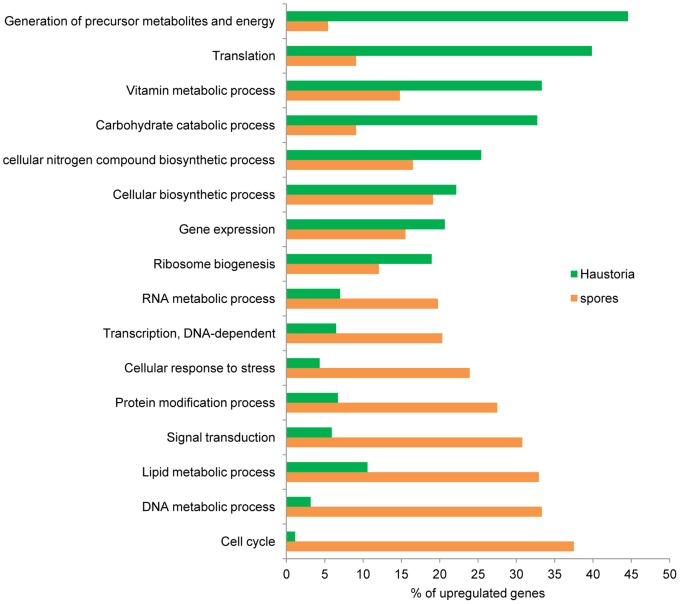
Comparative ontology analysis of transcripts with statistically significant changes in expression between haustoria and germinated spores. Of the original transcript set, 30.2% (601) of the 1,989 haustorial-enriched genes and 47% (1,109) of the 2,357 genes up-regulated in germinated spores were annotated with B2G. Relevant biological process GO terms are shown on the Y-axis. Percentages of genes differentially expressed in each tissue belonging to the nominated categories are shown on the X-axis.

**Figure 4:**
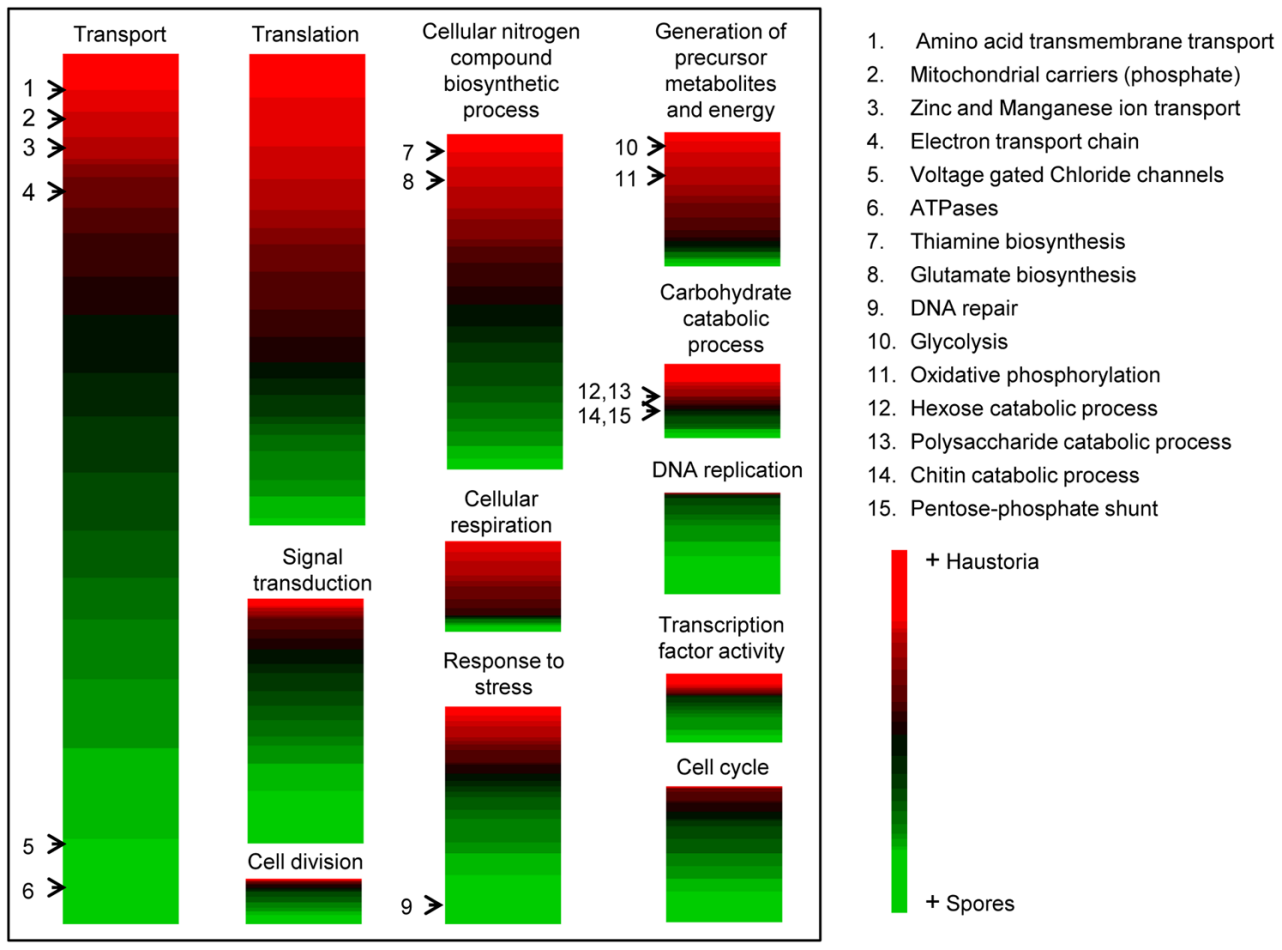
Heatmaps representing relative levels of gene expression classified by metabolic categories. Relevant categories were selected from the B2G analysis. Genes belonging to these categories were listed and organized according to transcript expression values (RPKMs) in haustoria and spores. The colours were based on haustorial expression relative to the overall expression in both tissues; red indicates high expression in haustoria and green, expression in germinated spores. Specific subcategories are shown by the black arrows indicating the presence of specific transcripts discussed in the text.

The transcripts in the reference set were categorised into functional classes using BLAST2GO to identify genes that encode proteins with known roles in cellular processes. Of this set, 4,485 transcripts could be unambiguously annotated. Using the list of the genes previously found to be differentially expressed, Fisher’s exact test was applied to find functional categories over-represented in either developmental stage. The major functional categories are shown in Figures 3 and 4. Broadly speaking, processes up-regulated in germinated spores were representative of cell proliferation, such as cell cycle control and DNA and cell wall metabolism, whereas haustoria were more engaged in energy production and biosynthetic processes. Figure 3 refers to transcripts overexpressed in each tissue in the selected GO categories, whereas Figure 4 includes all transcripts classified under the selected GO categories and relative levels of expression were represented as colours. This is important because processes included within the same ontogenic category can have fundamentally opposed activities. We discuss the data in more detail with reference to fungal development and metabolism, below.

#### DNA replication and cell cycle

The two sampled tissues represent very different developmental stages, with germinated spores involved in growth and division, whereas the haustorial stage is terminally differentiated. Generally, transcripts classified into the categories of cell cycle, DNA replication and cell division were more highly represented in germinated spores (Figure 4). Genes involved in DNA replication and cell division included DNA polymerase subunits and replication factors. Cell division genes were clearly enriched in germinated spores, consistent with the idea that haustoria are non-dividing fully differentiated cells. For example, cyclins (proteins that regulate cyclin dependent kinases (CDKs) to control cell division), cyclin-dependent kinases, cohesins (protein complexes that regulate the separation of sister chromatids during cell division), septins (GTP binding proteins that provide structural support during cell division and compartmentalize parts of the cell) and genes associated with the control of mitotic phase progression were all up-regulated in germinated spores (Figure 4 and Table S6, HeatMap). Although the expression of these genes makes no implication about their function *in vivo*, because both positive and negative roles in control of cell cycle are possible, the observations suggest that active cell cycle regulation occurs in germinated spores. One of the most interesting transcripts found to be up-regulated in germinated spores encoded a protein similar to the cyclin-dependent kinase Cdk5 (PST79_11595). Flor-Parra *et al.* [43] found that the cyclin Pcl12 represents a polarity- and virulence-specific regulator in *Ustilago maydis*, complexing with the cyclin-dependent kinase Cdk5 for sustained polar growth [43]. Although we found no *Pcl12* homologs in our data (nor in the *Pst* 130 draft genome [32]), *Pst* encodes other cyclin-like proteins that might regulate Cdk5 function. Homologs of *U. maydis* cyclin genes *B1* and *B2* were also found to be highly expressed in *Pst* germinated spores, with the latter one of the most strongly up-regulated genes of this category. The *U. maydis* genes are important in cell cycle regulation [44], with mutants arrested after S phase, while overexpression of cyclin B2 generates cells with anomalous DNA content and premature entry into mitosis. In *M. oryzae,* degradation of the homologous cyclin B proteins, CYC1 and CYC2, is necessary for mitosis exit and pathogenesis [45]. The connections between cell cycle, morphogenesis and virulence seem to be important for this pathogen to colonise plant tissue, and this may also be the case for rust pathogens.

Four transcripts with similarity to septin genes (PST79_4650, PST79_4652, PST79_4126, PST79_582) were found in our data, three of which were up-regulated in germinated spores. Septins constitute a cytoskeletal structure that is conserved in eukaryotes [46]. In *Saccharomyces cerevisiae*, septins assemble as a ring that marks the cytokinetic plane throughout the budding cycle and this structure participates in different aspects of morphogenesis, such as cell polarity, localization of chitin synthesis, and the spatial regulation of septation [46]. Recently Dagdas et al. [47] showed that septin proteins in *M. oryzae* assemble in a ring-shaped structure at the base of the appressorium together with F-actin and other cytoskeletal components to provide rigidity and negative membrane curvature for protrusion of the penetration peg into the host. Although *Pst* often can form appressoria over stomata prior to leaf penetration, this is not strictly necessary as penetration can often occur without appresoria development. Thus the role of septins in leaf penetration remains to be established.

#### Cell wall modification enzymes

Cell walls are complex polysaccharide structures composed mainly of cellulose, hemicellulose and pectin in plants [48], and chitin, glucans and other minor components in fungi [49]. Glycoside hydrolases (GH), polysaccharide lyases, and esterases are enzymes that allow formation, remodelling or degradation of cell walls and play a fundamental role in plant-fungal pathogen systems [50]. The battery of carbohydrate-modifying enzymes derived from plant pathogenic fungi can vary enormously according with the ecological niches occupied by different species. Recent genomic studies on plant fungal pathogens have shown that the number of GH genes correlates with the type of host interaction [23], [24], [51], [52], [53]. Biotrophic fungi seem to possess a reduced number of GH enzymes, consistent with the necessity of minimizing host cell wall damage to avoid triggering plant immunity [23], [24]. We found a total of 53 transcripts with similarity to GHs belonging to 18 different families that were expressed in *Pst* (Table S3, General metabolism). The most abundant families were GH5, GH18 and GH47, which agrees with recent findings for *Pgt* and *Mlp*, where the GH5 and GH47 families were expanded [23]. Thirteen *Pst* GHs were similar to enzymes implicated in plant cell wall degradation and interestingly, of these two α-galactosidases (PST79_3645, PST79_3417) and three β-mannosidases (PST79_2043, PST79_8128, PST79_4682) were up-regulated in haustoria. This suggests a requirement for GHs for entry into plant tissue, or alternatively, a role in haustorial wall maintenance. The higher expression of mannosidases in advanced stages of the infection has been reported in other rusts [23]. Moreover, two glucanases from family 16 (PST79_11351, PST79_11352) were massively up-regulated in germinated spores, which may have implications for host invasion. In barley, alpha-galactosidase activity is essential during leaf development by loosening cell walls and in cell wall expansion [54]. Thus, the higher expression of these enzymes may suggest that this is a fungal strategy to impede reinforcement of the host cell wall at the site of haustorial interaction. Additionally, eight *Pst* transcripts encoded proteins similar to β-1,3-glucanases, four of which were up-regulated in germinated spores (PST79_5045, PST79_2573, PST79_9378, PST79_8761) and one in haustoria (PST79_5049). These could potentially play roles either in fungal cell wall remodelling or degradation of plant callose. The remaining GHs were similar to enzymes required for glucan synthesis and fungal cell wall remodeling (almost all up-regulated in germinated spores), posttranscriptional modification of cell wall proteins, chitinases (almost all up-regulated in haustoria) and glycogen breakdown.

Related to cell wall modification, we also found genes with strong similarity to chitin synthases (mostly up-regulated in germinated spores) and carbohydrate deacetylases (including chitin deacetylases), which were massively up-regulated in germinated spores. Chitin deacetylases are of particular interest because they convert chitin into the less rigid chitosan, potentially avoiding recognition by plant chitin receptors [55]. The genomes of *Pgt* and *Mlp* [23] as well as the secretome of the symbiont *Laccaria bicolor* [56], were also found to be enriched in chitin deacetylase genes. Expression profiling of the biotrophic stage of the coffee rust *Hemileia vastatrix* suggested that two chitin deacetylases were most strongly expressed in the early stages of host invasion, coinciding with spore germination and tissue invasion [57]. Likewise, the rust fungus *Uromyces viciae-fabae* exhibits massive chitin deacetylase activity when the fungus starts to penetrate through the stomata [58]. An overall view of the data suggests complex regulation of GHs during fungal development to achieve a balance between the degradation of plant cell wall polysaccharides without triggering an immune response, and remodeling of the fungal cell wall via both degradation and synthesis to allow growth and development.

#### Transport proteins

Nutrient acquisition is presumed to be a major role of haustoria in rust infection. We therefore searched for potential nutrient transporters by BLASTx of the *Pst* contigs set against the NCBI non-redundant protein and the Transport Classification databases (TCDB: http://www.tcdb.org). The initial cut off for evaluation was e-value <10^−25^, and the hits were further annotated manually to remove alignments of less than 100 amino acid residues. The major transporter classes that we found constituted those for amino acids and oligopeptides, sugars, small molecules and ions, and the vitamin co-factor nicotinic acid. Some of these were expressed differentially in a manner that gives insight to the pathogenic strategy of the fungus (Table S4, Transporters).

#### Amino acid and oligopeptide transporters

Pathogenic fungi have a large requirement for nitrogenous compounds for macromolecule biosynthesis. The best characterized haustorial amino acid transporters are from *U. fabae*: AAT1p [59], AAT2p [22] and AAT3p [60]. In our data we found very close homologs to all three transporters (PST79_2586, PST79_1706, PST79_2986), each of which were expressed at very low levels in germinated spores, but expressed highly in haustoria, especially the *AAT2* homolog. We also found three other putative amino acid transporters, one highly similar to *AAT3* (PST79_3074) that was expressed strongly in germinated spores but at low levels in haustoria. Similarly to *U. fabae* [16], our data suggest that most amino acid uptake occurs via haustoria, but the high expression of one amino acid transporter in germinated spores might suggest that uptake can also occur from other cell types such as infective hyphae. It would be very interesting to test the specificity and affinity of *Pst* putative amino acid transporters; presumably the early expressed transporter should show very high affinity because of the scarcity of nutrients in the apoplast. In addition to this, eight predicted oligopeptide transporters (OPT) were found in our data, three up-regulated in haustoria (PST79_9468, PST79_1656, PST79_8271), one in germinated spores (PST79_2988) and the other four did not show expression biases. The amplification of the *OPT* gene family in *Pgt* and *Mlp* was proposed to be a genomic adaptation of these pathogens to obtain amino acids, nitrogen and carbon from oligopeptides derived from their host [23]. Overall our data suggest that haustoria are more active than germinated spores in uptake of amino acid compounds, although the early expression of some of those transporters could be crucial for the pathogen’s development.

#### ABC and MFS transporters

ABC transporters constitute a large superfamily of primary active transport systems that are present in all kingdoms of life, and play diverse physiological roles in trafficking of a wide range of substrates across internal and external membranes. These transporters represent one of the largest and most ancient transporter classes and derive their name from the shared highly conserved domain, the ATP binding cassette (ABC), which binds and hydrolyzes ATP [61]. MFS transporters are an ancient class of single-polypeptide secondary carriers which transport small solutes in response to chemiosmotic ion gradients [62]. We identified twelve transcripts encoding proteins with similarity to ABC transporters in the *Pst* transcriptome, but only three of these showed differential expression (PST79_11360, PST79_10409, PST79_132, up-regulated in germinated spores), so no generalizations about biological function were possible. In other biotrophic pathogens such as *Blumeria graminis* f.sp. *hordei*, a marked reduction in the number of genome-encoded ABC transporters (only 20) has been correlated with the loss of secondary metabolic enzymes, reported as the lowest number known in fungi [24]. In contrast, ABC transporters seem to play an important role during pathogenesis for the hemibiotroph *M. oryzae*, especially in the appressorial stage where far more ABC transporters are expressed than in any of the studied stages of *Pst* [63], [64], [65]. This may reflect a need for the pathogen to exclude small molecule defense compounds secreted by the host, and to deploy secondary metabolites during tissue colonization [65]. We also identified twenty six transcripts annotated as MFS-like transporters, 16 with putative substrates, and ten classified as general MFS transporters. Of the general MFS transporters, six were expressed preferentially in germinated spores (PST79_7841, PST79_11441, PST79_3438, PST79_8377, PST79_3969, PST79_87060), and two in haustoria (PST79_96, PST79_7181). Duplessis et.al [23] reported 51 and 88 MFS transporter genes in the *Pgt* and *Mlp* genomes respectively, however while the total number of such transporters in the *Pst* genome is presumably similar, only a few of them were expressed during the developmental stages and sampling conditions studied here. Nevertheless, most of the MFS transporters in *Pst* were preferentially expressed in germinated spores, which is the first infectious stage that encounters host defence. This could suggest that although rust pathogens are also equipped to export toxins or metabolites, the most important roles of these proteins is in growth and development.

#### Sugar transporters

Three putative glucose transporters (MFS), seven unspecified sugar transporters (MFS), one mannose transporter and one oligosaccharide translocation protein were identified in our set of transcripts. One of the transcripts classified as a glucose transporter in *Pst* (PST79_113) shares 92% similarity at the amino acid level with the hexose transporter HXT1p of *U.fabae* [14]. This gene showed the highest haustorial expression of all of the sugar transporters identified, and similarly to *Uf*-HXT1, showed almost no expression in germinated spores. The expression pattern of *Uf*-HXT1p is very similar to the amino acid transporter *Uf*-AAT2p [14]. Interestingly, the haustorial expression values for the *Pst* homologs of these two transporters were also almost identical, suggesting that expression of these genes is coordinated across different rust species. We also identified three *Pst* homologs of the H^+^-ATPase transporter *Uf-PMA1* (PST79_1028, PST79_343, PST79_4481) which is believed to be important for establishment of a proton gradient for coupling substrate translocation into haustoria [66]. Similarly to *Uf*-PMA1, all of the *Pst* homologs were expressed more strongly in germinated spores than haustoria [67]. Biochemical characterization of this *U. fabae* ATPase showed that despite the higher transcript levels in germinated spores than in haustoria, its enzymatic activity is far greater in haustoria where it may act as a proton-substrate symport mechanism for *Uf*-HXT1p [67], [68]. Thus, this mechanism may be conserved in *Pst* and potentially outlines a conserved transport mechanism for glucose that is key to the biotrophic lifestyle of rusts.

The remaining two putative glucose transporters (PST79_2057, PST79_2930) were up-regulated in germinated spores, suggesting that the fungus prepares for tissue invasion and is poised to exploit host carbohydrates as a nutritional source, or alternatively that these could export substrates for cell wall synthesis. *M. oryzae* expresses sugar transporters at an analogous stage; during appresoria formation but before tissue penetration, supporting our reasoning [65]. Finally, of the seven unspecified sugar transporters, three were up-regulated in haustoria and three were more strongly expressed in germinated spores. One of the genes expressed in germinated spores (PST79_2314) has a high similarity to STL1 (e-val 1×10^−101^ with the *Saccharomyces cereviseae* gene), a glycerol-proton symporter that is inactivated by glucose [69]. This might suggest that glycerol could be a primary carbon source, or alternatively plays a role as an osmoprotectant during spore germination, as has been shown for other fungi [70], [71], [72], [73].

#### Phosphate transporters

Inorganic phosphate is an essential nutrient required for the synthesis of nucleic acids, phospholipids, cellular metabolites, and protein modification, reactions that require high phosphate concentrations [74]. We identified four transcripts encoding different phosphate transporters from our dataset. One of these was annotated as the phosphate translocator of the inner mitochondria membrane, discussed below. From the remaining three, two were up-regulated in germinated spores (PST79_3467, PST79_11745) and annotated as inorganic phosphate transporters related to Pho84, a phosphate starvation-inducible high-affinity H^+^/PO_4_^3−^ symporter [75]. The third transporter (PST79_8877) was annotated as an SPX-domain containing protein, a domain often present in proteins involved in regulating inorganic phosphate homeostasis [76]. In budding *Saccharomyces cerevisiae* cells, response to phosphate starvation includes the up-regulation of the activity of a high-affinity phosphate uptake system [77]. Pho84p is one of the key proteins in this phosphate transport system, which is regulated transcriptionally in response to extracellular phosphate levels sensed by a phosphate-responsive signal transduction pathway [78]. The expression of this gene is greatly induced in low-phosphate medium and is essential for growth under low-phosphate conditions [75]. The presence of a homologous gene in *Pst* and its up-regulation in germinated spores could suggest that similar phosphate starvation sensing/signalling mechanisms exist in rusts, which could be crucial for cell cycle progression and growth as has been shown in yeast [79]. In addition, thirteen transcripts similar to putative ion transporters with specificities for Ca^2+^, Cu^2+^, Fe^2+^, Mg^2+^, Mn^2+^, K^+^ and Zn^2+^ were identified in our *Pst* data. Transporters for Zn^2+^ and Mn^2+^ were up-regulated in haustoria, while transporters for K^+^ and Ca^2+^ were up-regulated in germinated spores.

#### Nicotinic acid transporters

Nicotinic acid is an essential cofactor for many enzymic processes. We found four transcripts (PST79_362, PST79_7845, PST79_4348, PST79_3107) encoding proteins with similarities to the *S. cereviseae* nicotinic acid transporter TNA1 (e-value ∼1×10^−30^) [80] in our data, one of them notably up-regulated in haustoria. Vitamin B3 (nicotinamide and nicotinic acid) is essential to living cells because it can be converted to nicotinamide adenine dinucleotide (NAD^+^), which can be reduced to NADH or the phosphorylated form NADPH. Generally, NADPH is a reductive intermediate for biosynthesis pathways, whereas NADH is an energy precursor and protects against oxidative damage. Thus uptake of host-derived niacin could be important in the haustorial stage which is greatly involved in energy production, biosynthesis of molecules for spore formation, and protection against oxidative stresses. Duplessis et al. [23] reported the *in planta* expression of four *Mlp* genes with similarity to *S. cereviseae* TNA1 permease at 96 hpi, supporting the idea that uptake of host nicotinic acid plays an important role during the advanced stages of the infection. We also searched for contigs that encode enzymes required for *de novo* synthesis of niacin from tryptophan. The known pathway in organisms that use tryptophan as a source for NAD^+^ (including yeasts, humans and some bacteria) involves six enzymes; we were able to identify five of these in our dataset, two of which were up-regulated in haustoria (Table S3, General metabolism). Thus it remains an open question whether *Pst* can synthesize niacin *de novo*, or it relies exclusively on its host for obtaining this essential cofactor.

#### Energy use – oxidative phosphorylation

**Figure 5:**
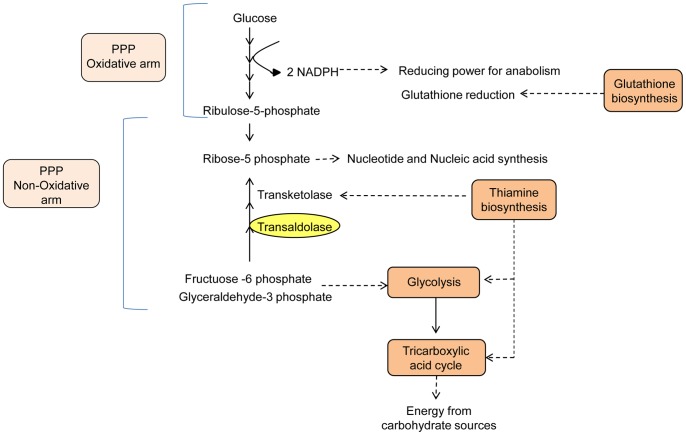
Metabolic processes in haustoria. Metabolic processes or specific enzymatic activities overrepresented in haustoria identified by B2G analysis and manual annotation are highlighted in this cartoon. Orange boxes and yellow ovals are metabolic processes or particular enzymes that showed statiscally significant upregulation in haustoria. Light orange boxes are metabolic processes where some genes showed statiscally significant upregulation in haustoria, and others showed a tendency to be more expressed in haustoria but were not statistically significant.

Transcripts classified in this category were represented more in haustoria than in germinated spores (Figures 3 and 4). Transcripts with similarity to genes involved in primary pathways of energy production comprising glycolysis, citric acid cycle, and oxidative phosphorylation were mostly up-regulated in haustoria (Table S5, Energy metabolism). Similar observations have been made for the obligate biotrophs *U. fabae* [81] and *Blumeria graminis* [82] where genes related to glycolysis are up-regulated during the parasitic stage. Those few *Pst* genes with higher expression in germinated spores corresponded to the key enzymes of the glyoxalate cycle, which will be described later. In addition, a phosphate translocator of the inner mitochondria membrane (PST79_4938), which carries out the coupled transport of H_2_P0_4_^−^ and H^+^ for subsequent ATP synthesis via the electron transport chain, was massively up-regulated in haustoria, consistent with a high demand for ATP in this tissue. This could be a consequence of the different nutritional status of haustoria and germinated spores. While the stored energy resources are limited in spores, haustoria can derive sugars abundantly from the host to feed energy production pathways and drive biosynthetic pathways for subsequent development (Figure 5).

#### Fatty acid metabolism

**Figure 6:**
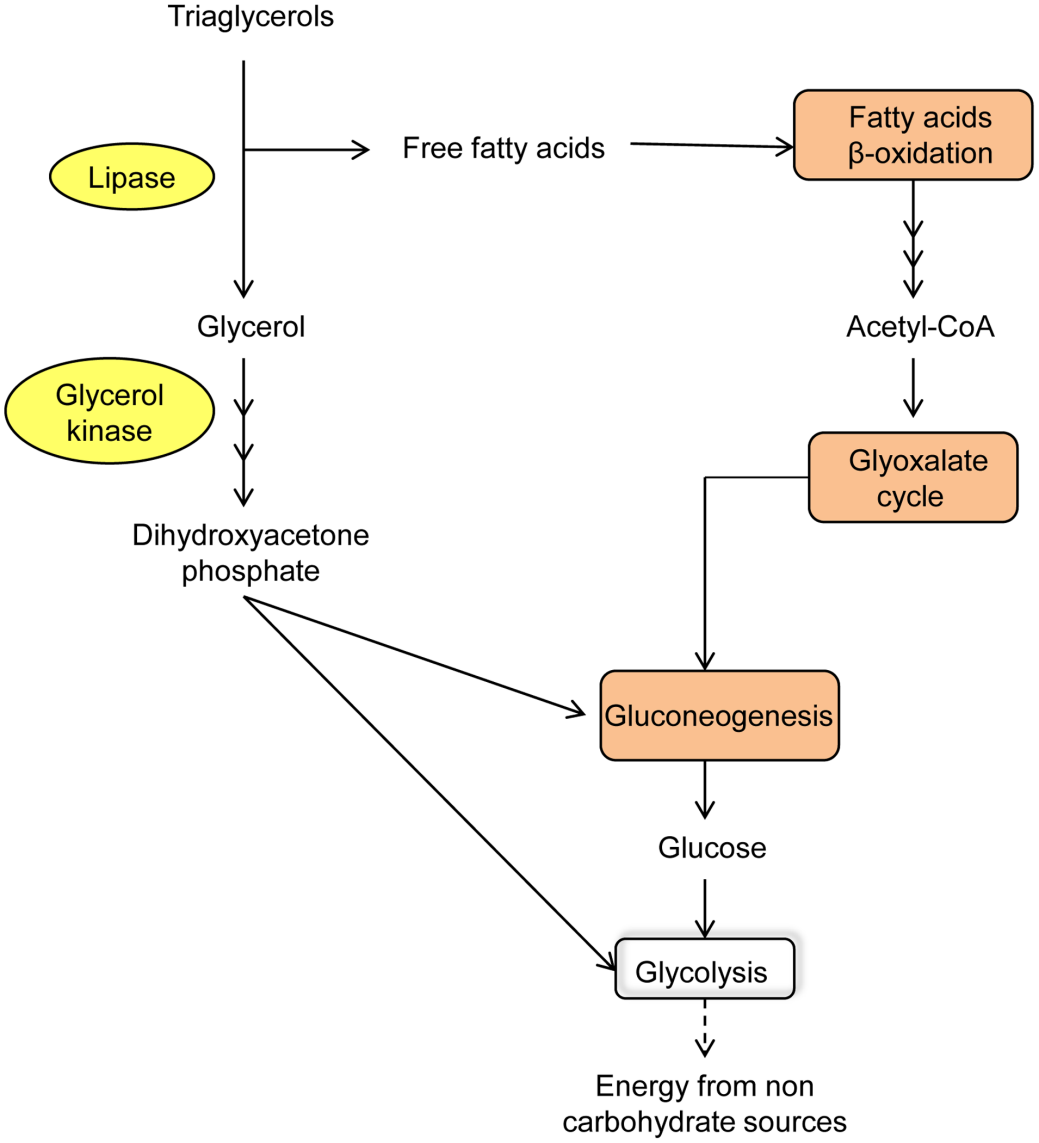
Metabolic processes in germinated spores. Metabolic processes or specific enzymatic activities overrepresented in spores identified by B2G analysis and manual annotation are highlighted in this cartoon. Orange boxes and yellow ovals are metabolic processes or particular enzymes that showed statistically significant upregulation in germinated spores.

Acetyl-CoA is the basic currency of carbon metabolism within the cell. It is the immediate product of carbohydrate catabolism and β-oxidation of fatty acids, and when in excess, can be used for biosynthetic processes through the glyoxylate cycle, gluconeogenesis and glyceroneogenesis pathways. In our transcriptomic study, the gene for triacylglycerol lipase (PST79_8009) together with nearly all those for the enzymes of glyoxylate cycle, gluconeogenesis and glyceroneogenesis were significantly up-regulated in germinated spores compared with haustoria (Table S3, General metabolism). This suggests that the activity of metabolic pathways for converting fatty acids into carbohydrates is important in the initial stages of infection. Key genes involved in β-oxidation of fatty acids such acyl-CoA dehydrogenase (PST79_1352) and enoyl-CoA hydratase (PST79_4902) were also up-regulated in germinated spores, suggesting that once triaglycerides are broken down, the released fatty acids are oxidised and the acetyl-CoA produced can be fed into gluconeogenesis by means of the glyoxylate cycle. The two key enzymes in the glyoxylate cycle, isocitrate lyase (PST79_6444) and malate synthase (PST79_4401), were highly up-regulated in germinated spores. However, malate dehydrogenase and citrate synthase, two other enzymes in this cycle, did not show the same biased expression pattern between the two tissue types. Malate dehydrogenase participates in the glyoxylate cycle and is also involved in the malate-aspartate shuttle, which is the system responsible for translocating electrons produced in glycolysis through the mitochondrial membrane for oxidative phosphorylation. Thus, the role of this enzyme is expected to be important in germinated spores but also in haustoria because of their high glycolytic activity. Apart from fatty acids, the second product of the triaglycerol breakdown is glycerol. This compound can be converted to intermediates of gluconeogenesis by means of glycerol kinase (PST79_6140) and glycerol-3-phosphate dehydrogenase (PST79_3414), genes which also showed significant upregulation in germinated spores. As mentioned previously, a gene similar to *STL1* (encoding a glycerol transporter) was also up-regulated in germinated spores, which suggests that glycerol derived from the host apoplast could be taken up and incorporated via the gluconeogenic pathway. Finally, genes encoding enzymes involved in glycogen breakdown were also up-regulated in germinated spores, which also results in glycerol production. In *Pgt* uredinospores, as well as spores of other plant fungal pathogens, glycogen is a stored energy source that is utilized during germ tube extension [83], [84]. These observations are consistent with data from other biotrophic and hemibiotrophic fungi such as *B. graminis* and *M. oryzae* [65], [82], where similar metabolic activity has been detected in early stages of the infection. Overall, our results point towards spore germination utilising stored compounds including lipids and glycogen as sources for energy production (Figure 6), whereas the massive upregulation of *HXT1* and enzymes of glycolysis, TCA cycle and oxidative phosphorylation in haustoria suggest that host-derived glucose is the primary carbon source.

#### Pentose Phosphate Shunt (PPS)

The PPS is an alternative fate for glucose which produces NADPH and ribose-5-phosphate. Ribose-5-phosphate can be used for nucleotide synthesis (oxidative branch), or recycled for energy production via glycolysis (non-oxidative branch). An important product of the PPS is the reductant NADPH, which is used almost exclusively in anabolic pathways, for example biosynthesis of fatty acids, amino acids and nucleotides including ATP. We found evidence for expression of all enzymes of the PPS in both germinated spores and haustorial (Table S3, General metabolism). Interestingly, all of the enzymes participating in the oxidative branch of this pathway showed an expression bias in haustoria as did transaldolase, which belongs to the non-oxidative branch. In germinated spores, the genes encoding the enzymes ribulose-phosphate 3-epimerase (PST79_10575) and phosphoglucomutase (PST79_5659) were up-regulated. The ability of this pathway to work in different directions suggests that it could play different roles in each tissue type. While both tissues are expected to have a strong requirement for biosynthetic processes, this is expected to be higher in haustoria which presumably contribute to provision and transport of substrates to fuel the growing infection and eventual spore biogenesis (Figure 5). Thus, the haustorial PPS seems to engage the oxidative arm for maximal NADP^+^ reduction and ribose-5-phosphate production. Additionally, an important enzyme of the non-oxidative branch, transaldolase, showed higher expression in haustoria than in germinated spores. The non-oxidative branch can convert five carbon sugars into six and three carbon sugars, which are fed into glycolysis. This would appear to be unnecessary in haustoria which should not be limited for glucose. Alternatively, the non-oxidative arm of PPS may work in the opposite direction, taking fructose-6-phosphate and glyceraldehyde-3-phosphate generated by glycolysis to produce ribose-5-phosphate, which is the basis of production of ATP, NAD^+^ and nucleic acids. However, the remainder of the enzymes that participate in the non-oxidative arm did not show a clear expression trend, which precludes clear conclusions on the role of this branch of the PPS. In *Plasmodium falciparum*, a human pathogen with an intracellular niche that is comparable to the rust haustorial stage, the oxidative arm is thought to operate during early stages of parasite development while the non-oxidative arm is active later in the cycle [85], [86]. These studies also showed that transcription of the PPS genes is not always coordinated, with activity of the pathway as a whole often dependent on expression of a single gene whose transcription is the rate limiting step for deployment of the pathway’s activity. In *B. graminis,* the absence of clear coordination in expression of the enzymes of PPS has also been observed [82]. These discrepancies require direct biochemical investigation which will provide more clues about the physiological implications of these patterns.

#### Thiamine biosynthesis

A number of metabolic enzymes require the vitamin B1 derivative thiamine pyrophosphate as a cofactor. Examples from the pathways described here include alpha–ketoglutarate dehydrogenase (α–KGDH) in the TCA cycle; transketolase in the non-oxidative PPS; and pyruvate dehydrogenase (PDH) which connects glycolysis and the TCA cycle. We found that transcripts associated with the thiamine biosynthesis pathway were massively up-regulated in haustoria, with the exception of the genes for thiamine-phosphate kinase and thiamine pyrophosphokinase which could not be found in our data. These enzymes are responsible for phosphorylating thiamine phosphate and thiamine diphosphate respectively, and interestingly, the latter is also absent in the genome of the malarial parasite *P. falciparum* [85]. The genes we found include those encoding homologs of thiamine biosynthesis genes *THI1* (4-amino-5-hydroxymethyl-2-methylpyrimidine phosphate synthase)(PST79_1423, PST79_612, PST79_1422) and *TH2* (hydroxyethylthiazole phosphate synthase)(PST79_882) from *U. fabae* identified previously by Hahn and Mendgen [13]. The massive expression of these genes almost exclusively in haustoria agrees with studies done in other rusts [23], [28], but it is not clear why the pathogen requires such high expression at this stage, which seems excessive if the role is simply to produce sufficient cofactors for metabolic enzymes. One explanation may be that thiamine is exported to other tissues. Alternatively, thiamine is known to alleviate stress in different organisms as an antioxidant. It is interesting that in the oomycetes, all haustorium-forming species have lost the thiamine biosynthetic pathway [87], which suggests that they must obtain thiamine from the host.

#### Nitrogen metabolism

Previous genome studies of obligately biotrophic fungal plant pathogens have noted the absence of genes encoding enzymes for nitrate and sulfur assimilation [23], [24]. Likewise, we were unable to identify genes for nitrate reductase or nitrite reductase, or for a nitrate transporter that could import nitrate directly from host tissue. Instead, we found a transcript encoding a protein with very high similarity to an ammonium transporter (PST79_5204) expressed in both germinated spores and haustoria. Ammonium is the preferred form of nitrogen for most organisms [88] and is assimilated into glutamate and glutamine. Glutamate synthase and glutamine synthetase are the key enzymes involved in ammonia assimilation. Glutamate is formed from α-ketoglutarate and glutamine in a reaction that is catalyzed by glutamate synthetase, followed by amination of glutamate by NH4^+^ to form glutamine catalyzed by glutamine synthetase. Subsequently, glutamate is used to synthesise the majority of amino acids, whereas glutamine serves as an amino-donor during other biosynthetic processes. Interestingly, the transcripts encoding genes for both glutamine (PST79_3032) and glutamate synthase (PST79_2575) identified here were up-regulated in haustoria, consistent with a greater role for biosynthesis in this organ. Genes encoding other enzymes that participate in subsequent steps including glutamate dehydrogenase (PST79_536), aspartate aminotransferase (PST79_7929), asparagine synthase (PST79_1116) and asparaginase (PST79_83), were all expressed in both tissues, suggesting nitrogen compounds obtained from the host can be converted freely into compounds required for pathogen growth and development.

#### Sulfur metabolism

Sulfur is an essential component of living cells as it is a fundamental component of the amino acids methionine and cysteine, Coenzyme A, and iron-sulfur enzymes. Most fungi take up sulfur as sulfate which is reduced to sulfide as a precursor of cysteine [89]. Despite this, the steps for sulfate assimilation are energetically expensive and fungi prefer to utilise cysteine or methione. To date, enzymes for sulfate uptake and reduction have not been identified in obligate biotrophs such as *Hyaloperonospora arabidopsidis* [90], *B. graminis* [24], or *P. graminis* f.sp. *tritici* [23]. In addition, genes encoding enzymes for sulfate reduction including sulfite reductase and phosphoadenosine phosphosulfate reductase were absent, consistent with observations for other obligate plant pathogens. However, we found a contig (PST79_3132) that encodes a transporter with very high similarity to S-methylmethionine (SMM) permease, which was almost exclusively expressed in haustoria compared to germinated spores. This is interesting because SMM is present at very high levels in wheat phloem [91] and as such, could be an abundant source of sulfur metabolites for the parasite. Apart from this, we were unable to identify candidate cysteine or methionine transporters in our data. Lastly, we identified a gene encoding cysteine synthase (PST79_10177), which assimilates reduced sulfur (S^2−^) into cysteine, expressed at low levels in both tissues. Genes with high similarities with those involved in the interconversion of homocysteine and cysteine through the intermediary formation of cystathionine (transsulfuration pathways) were also identified. This pathway creates cysteine from methionine.

#### Glutathione metabolism

Glutathione provides redox buffering to cells, and plays a role in many cellular processes including iron metabolism [92]. The anti-oxidant properties of glutathione contribute to anti-defence against host active oxygen compounds. Glutathione is derived from the amino acids glycine, glutamate and cysteine. The two key enzymes for synthesis of glutathione are glutamate-cysteine ligase (PST79_3565) and glutathione synthetase (PST79_1178).Both of these genes showed greater expression in haustoria, and PST79_3565 was significantly upregulated. This could suggest an ongoing need for antioxidants in the static haustorial stage which is exposed to plant defences. We also note that glutathione reductase (PST79_10658) and glutathione peroxidase (PST79_5949)(enzymes involved in the redox balance) as well as genes involved in the response to oxidative stress were more highly expressed in germinated spores than in haustoria (Table S3, General metabolism). All this would suggest the presence of glutathione in spores prior to development. Thus, one function of haustoria could be to synthesise glutathione for subsequent deposition into new spores as a pre-formed environmental defence.

#### Cytochrome P450s

Cytochrome P450s comprise a diverse superfamily of proteins containing a heme cofactor that catalyse the oxidation of organic substances. Among their most common substrates are steroidal molecules as well as drugs and toxins. The P450 system is important in fungal evolution for adaptation to ecological niches [93], and its potential role in compound detoxification including metabolism of plant defense molecules [13], [94]. A total of 29 and 17 P450s are encoded by the genomes of *M. larici-populina* and *P. graminis* f. sp. *tritici* [23] respectively. In the current dataset, we identified only eight expressed P450 genes, five of which were up-regulated in haustoria (Table S3, General metabolism). One of the P450s (PST79_8680) we identified is highly similar to CYP51A1, a lanosterol 14 alpha-demethylase, required for conversion of lanosterol to cholesterol. This enzyme is targeted by the commonly used azole class of antifungal drugs, which occupy the active site of the enzyme inhibiting the production of ergosterol, a component of the fungal cell membrane [95]. Two other P450s (PST79_1792, PST79_1793) up-regulated in haustoria belong to the CYP67 family, which was first reported in *U. fabae* as an in planta-induced gene [13]. The remaining genes encode unspecified mono-oxygenases.

#### Transcription factors

Overall, our analysis revealed approximately 37 transcripts encoding proteins related to transcription factor activity, from both haustoria and germinated spores (Figure 4 and Table S6, HeatMap). Despite this, it is difficult to obtain specific insight into the patterns and functions of these genes. However, there were a few exceptions where we could predict a possible function based on BLAST2GO and BLASTx searches (Table S3, General metabolism). One of the most interesting genes found in our data set was a *Ste12*-like gene (PST79_9215), which was up-regulated in haustoria. *Ste12* was first identified in a yeast sterile mutant, and multiple studies have shown that *Ste12*-like genes play major roles in regulating morphogenetic programs in response to environmental changes (see review [96]). Yeast *Ste12* is an important regulator of invasive growth and pseudohyphal development [97] and its homologs in a number of fungi are important for sexual development and pathogenicity [98], [99], [100], [101]. In the root pathogen *Fusarium oxysporum,* a *ste12* mutant was impaired in pathogenesis on tomato and the pathogen could not differentiate into specialized infectious structures [102]. In the hemibiotrophic fungal pathogens *Colletotrichum lagenarium*, *Colletotrichum lindemuthianum* and *M. oryzae, Ste12*-like genes are important for penetration of leaf surfaces from appresoria and subsequent invasive growth [100], [103], [104]. Moreover, some evidence suggests that *Ste12* plays a role in host penetration of the arbuscular mycorrhiza *Glomus intraradices* [105]. Although the role of Ste12 transcription factor in rust fungi is unknown, we suggest that the *Pst* homolog could play a role in specifying haustorial development.

A second interesting gene was the homolog of *TUP1* (PST79_2626), a conserved transcriptional regulator from fungi and mammals, which controls dimorphism in certain fungi [106], [107]. The change in morphology between yeast-like growth and a filamentous state in response to environmental signals is frequently associated with virulence in plant pathogenic fungi [106]. *TUP1* plays a central role in controlling the expression of the genes implicated in the genetic control of mating, filamentation, and pathogenic development of *Ustilago maydis* [108]. Analyzing the function of *TUP1* in rust fungi could provide a better understand of how it acts within the unique biological context in which these pathogens develop.

Lastly, five other genes associated with transcription factor activity were found in our data, encoding a putative CCAAT-box binding factor (PST79_5498), a putative activator of basal transcription (PST79_5377), an MCM-domain-containing protein (minichromosome maintenance ATPase) (PST79_3174), a putative bZIP transcription factor (PST79_8536) and a gene with similarity to the regulator *Cys3* (PST79_492) reported originally in *Neurospora crassa* [109]. *Cys3* controls the synthesis of a set of catabolic enzymes for utilization of secondary sulfur sources when sulfur-containing amino acids and inorganic sulfate are missing or limited [110]. The presence of a similar gene in *Pst* might suggest that secondary sulfur sources play a role in biotrophy, and upregulation of this gene in haustoria could suggest these sources are derived from the host.

#### Signal transduction

Cellular differentiation and filamentous growth are finely regulated in many fungi in response to environmental and nutritional signals. External stimuli must be transmitted within the fungus by signal transduction pathways to enable the appropriate response. Our transcriptomic analysis revealed high expression of signal transduction components in both germinated spores and haustoria. Transcripts encoding proteins related to two component response regulators, the Ras protein superfamily of guanine nucleotide exchange factors, and diverse protein kinases were most highly represented. In fungi, two-component signalling systems participate in processes such as environmental sensing, oxidative stress response, cell-cycle control, and switching between non-pathogenic and pathogenic states [111], [112], [113]. They are typically composed of a sensor-kinase protein, a phosphorelay protein, and a response regulator. We found genes encoding two-component regulatory proteins in both haustoria and germinated spores (Table S6, HeatMap). Generally, the proteins expressed in each tissue were distinct, consistent with the idea that they respond to different stimuli. There was a larger representation of these genes in germinated spores suggesting a more complex interpretation of environmental signals prior to development of the haustorial structure. One gene up-regulated in germinated spores (PST79_186) has high similarity to Os-1, a two-component histidine kinase originally isolated from *Neurospora crassa*. Os-1, also known as Nik-1, is required for adaptation to high osmolarity [114], [115]. Three groups of selective fungicides target the osmotic stress signal transduction pathway [116], [117]. Intracellular osmotic pressure plays important roles in fungal development, for example as inside the *M. oryzae* appresorium [118]. Although disruption of *HIK1* (*Os-1*) in this pathogen caused no defect in growth on normal media or in pathogenicity to rice plants, the mutant strain acquired resistance to three groups of fungicides (phenylpyrroles, dicarboximides, and aromatic hydrocarbons) [119]. The almost exclusive expression of this gene in *Pst* germinated spores suggests that the osmosensitive signal transduction pathway could play a role in the early stages of stripe rust disease.

Ras proteins are a superfamily of guanine nucleotide exchange factors in which the phosphorylation status of the bound nucleotide operates a binary switch. They have numerous signaling roles in cellular functions including cytoskeletal integrity, proliferation, differentiation, cell adhesion, and cell migration. Ras proteins are active in the GTP-bound form, which is promoted by guanine nucleotide exchange factors (GEF) in an exchange reaction with GDP, and deactivated by GTPase activating proteins (GAP) that promote dephosphorylation of the bound GTP nucleotide. We identified evidence of expression of genes encoding proteins with similarity to Ras proteins called ADP ribosylation factors (ARF) in both sampled tissues, which is of particular interest to rust pathogenesis because they regulate vesicular traffic, phospholipid metabolism and actin remodelling. Other genes encoding other types of Ras protein as well as GEFs and GAPs were also found in both tissues, suggesting complete representation of these complex molecular switches that regulate cell fate.

### Conclusions

Transcriptome studies such as the current work considerably enhance analysis of genome information. The 454 platform used here gives significantly longer reads than Illumina, which both helps with transcript assembly and demonstrates gene expression. In contrast, small-read based assemblies are subject to assembly errors and the uncertainties of gene prediction, although these also benefit from annotation with transcriptomic data. One important aspect of defining transcriptomes, as described here, is that it cuts through the complexity of the genome. Thus, while the predicted coding capacity of rust genomes is ∼22,000 genes, we identified ∼12,000 expressed contigs in the sampled tissues. Presumably, many of the remaining genes are expressed in different contexts (for example on the alternate host, or as part of different spore stages), or were simply undetected or unassembled in our study. Although isolation of haustoria is a very powerful way to track the pathogenic stage of the fungus, we did not analyse infected tissue which includes the infectious hyphal stage that presumably also secretes effector proteins. Another important aspect of our study is quantification of expression using Illumina data, which considerably enhances the accuracy and reliability of our expression measurements. Thus, use of 454 and Illumina together provides a robust expression data set. This allowed us to predict a suite of 437 potential effector genes, defined chiefly as genes upregulated in haustoria that encode secreted proteins. We also note that not all secreted proteins will be virulence effectors, and some within our dataset show clear hallmarks of roles in fungal cell wall modification.

The current data provide a comprehensive view of the stripe rust transcriptome in germinated spores and haustorial stages. Overall, the fungus seems to use similar biochemical pathways to those described in better characterised species such as *M. oryzae*, but with adaptation to the particular pathogenic lifecyle of wheat rusts. Haustoria and spores are clearly working in different ways. The germinating spores prioritises polarised growth from stored energy until such time as it is able to mine its own resources, whereas haustoria are a sessile stage devoted to nutrient extraction and defence suppression. The uredinospore contains vast stores of energy in the form of lipid bodies, sugar alcohols and proteins [120], [121] to allow the foraging germling to find the pathogenic niche, and in that sense is analogous to a plant seed. From our data, we can see that haustoria create abundant demand for sugars, amino acids, and nitrogen, through high expression of transporters for these fundamental building blocks. In addition, we propose a new mechanism for sulphur assimilation by uptake of methylmethionine. Sugars are used immediately by haustoria for the production of ATP through glycolysis, TCA cycle and oxidative phosphoryation. By contrast, spores seem to obtain energy by utilising lipid resources via the glyoxylate pathway. A further important haustorial function is biosynthesis of macromolecules, and it is clear that steady production of the reductant NADPH is made possible by import of nicotinic acid through dedicated transporters, and its ultimate conversion to NADPH by the PPS. Spores also use the PPS but it is likely that this is works in the opposite (oxidative) direction, for energy production. Molecules made in haustoria are presumably exported directly to spores for biogenesis and storage, so the molecular pathways active in haustoria should reflect spore composition directly. With respect to the completeness of our dataset, we note as have previous authors that many genes are novel so cannot be annotated. For this reason, it is difficult to exclude the presence of certain genes with major implications for the biotrophic lifecycle, such as N and S assimilation. Of course, the absence of these genes could also be ascribed to low expression of miss-assembly of small reads. These are important challenges for the field because barring technological breakthroughs, most fungal genome sequences are likely to have large components of small sequence reads in their genesis.

## Materials and Methods

### Strains and Culture Conditions

Seedlings of the *Pst*-susceptible wheat cultivar Morocco were grown in the greenhouse under 70% relative humidity at 21°C and 16∶8 light:dark cycle. Seven-day old seedlings were inoculated with fresh uredinospores of *P. striiformis* f.sp. *tritici* strain 104E137A- [1], and incubated for 48 h in 100% humidity at 9°C in the dark. Subsequently plants were transferred to a growth chamber at 17°C with a 16∶8 light cycle. About 20 grams of heavily infected tissue were collected 9 days after infection (dai; just prior to sporulation) and immediately processed for haustoria isolation. Germinated spores were obtained by germination of about 80 mg of fresh spores harvested from infected leaves 17 dai on sterile distilled water at 9°C for 15 h in the dark. Germinated spores were collected by filtration with an 11 µm nylon mesh, and frozen in liquid nitrogen and stored at −80 prior to extraction of total RNA.

### Haustoria Isolation

For 454-based transcriptome analysis, haustoria were isolated from stripe rust-infected Morocco leaves at 9 dai by Concanavalin A affinity chromatography (Text S1 and Figure S4) [122]. Fifteen separate preparations were made each from 20 g of infected wheat leaves that were sequentially washed with chilled tap water, 2% bleach, water, 70% ethanol and Milli-Q purified water. Haustorial isolation was performed as described [123] using a final 20 µm pore nylon mesh to remove the bulk of the plant cell material before affinity purification. Purified haustoria were pelleted by centrifugation at 14,000 *g* for 5 min, frozen in liquid nitrogen and store at −80°C prior to RNA isolation.

For Illumina RNAseq expression profiling, haustoria were purified by a Percoll gradient method. Twenty g of infected wheat leaves were treated as above, but after passing through the 20 µm mesh, the filtrate was centrifuged at 1080 *g* for 15 min at 4°C and the resulting pellet was resuspended in 20 ml of 1× isolation buffer [0.2 M sucrose, 20 mM MOPS pH 7.2] containing 30% Percoll. The suspension was divided into four tubes and centrifuged at 25,000 *g* for 30 min at 4°C. The first 10 ml of each tube was recovered, diluted 10 times with 1× isolation buffer and centrifuged at 1080 *g* for 15 min at 4°C. The pellets were resuspended in 20 ml of 1× isolation buffer containing 25% Percoll and centrifuged at 25,000 *g* for 30 min at 4°C. The first 10 ml of each tube was recovered, diluted 10 times with 1× isolation buffer and centrifuged at 1080 *g* for 15 min at 4°C. The final pellet was frozen in liquid nitrogen and stored at −80°C prior to RNA isolation.

### Light Microscopy

Bright field and fluorescence images of haustoria isolated by affinity chromatography were collected on a Leica DMR epifluorescence microscope using a 40× objective. Cells were labelled with 100 µg ml^−1^ FITC-conjugated wheatgerm agglutinin (Sigma) or Concanavalin A, Alexa Fluor 594 conjugate 10 µM (Invitrogen), by incubating them for 30 min at room temperature in phosphate-buffered saline. Viability tests were done on ultrapure haustoria isolated on Percoll gradients combined with flow cytometry (Garnica & Rathjen, manuscript submitted) by using CellTracker™ Orange CMRA (Invitrogen) following the manufacturer’s instructions.

### Isolation and Sequencing of mRNA

Frozen haustoria and germinated spores samples were ground in liquid nitrogen, and total RNA was isolated using the QIAGEN (Doncaster Australia) Plant RNeasy kit following the manufacturer’s instructions. RNA was eluted in RNase-free water and checked for integrity on an Agilent 2100 Bioanalyzer. For 454 sequencing, approximately 5 µg of total RNA from isolated haustoria and 20 µg of total RNA from germinated spores were used to isolate mRNA using Dynabeads Oligo(dT)_18_ (Dynal, Norway) according to the manufacturer’s instructions. cDNA was produced and fragmented by nebulization, and 454-GS-FLX titanium pyrosequencing (single-end strategy) was carried out on a Roche 454 Titanium sequencer at the Biomolecular Resource Facility (Australian National University, Canberra, Australia). Half a plate was used following standard procedures recommended by Roche. Initial quality filtering of the raw reads was performed using Roche proprietary analysis software Newbler (software release 2.0.00.22) to remove poor quality reads and adapter sequences.

For Illumina sequencing, approximately 10 µg of total RNA per biological replicate of isolated haustoria and germinated spores were processed with the mRNA-Seq Sample Preparation kit from Illumina to produce the sequencing libraries. Quality and quantity controls were done on Agilent 2100 Bioanalyzer using a DNA 1000 chip kit and each library was diluted and used for sequencing using an Illumina Genome Analyser GX II platform. Libraries from haustoria samples and germinated spores samples were sequenced with 100-base paired-end reads.

### Whole Transcriptome Analysis by 454 and Illumina Sequencing

CLC Genomic Workbench 4.0 software (http://www.clcbio.com/) was used for *de novo* assembly of 454 reads, prediction of ORFs, Illumina read mapping against reference contigs, computation of normalized counts (expressed in reads per kilobase of exon model per Million mapped reads – RPKM [41]) and differential expression analysis. The parameters used for *de novo* assembly were similarity 0.97; length fraction 0.5; insertion cost 3; deletion cost 3; mismatch cost 2. The mapping of Illumina reads against reference contigs was done after removing adapter sequences and reads of low quality (trim quality score 0.05, max nucleotide ambiguities 2 and minimum number of nucleotides in reads = 35), allowing up to two base mismatches. Reads that mapped to multiple sites were assigned randomly and proportionally to one of the mapped sites. The random distribution was done proportionally to the number of unique matches that the genes to which it matches have, normalized by the transcript length.

Three biological replicates of each tissue were used for differential gene expression analysis. A 0.84–0.88 Pearson correlation coefficient was obtained for haustoria biological replicates and 0.88–0.93 for germinated spores biological replicates. The differential expression between haustoria and germinated spores samples was evaluated using Baggerley’s test [42] by treating the same types of sample as one group. The genes with a false discovery rate (FDR) corrected *P*-value less than 0.05, a fold change >2 and a difference of at least 20 were considered to be significant. Short reads for the 454 and Illumina datasets were deposited in the NCBI Sequence Read Archive under the accession numbers SRR579533-40.

For the gene ontology (GO) classification the reference transcripts set created by pooling raw reads from both transcriptomes was analyzed using BLAST2GO 2.5.1 [36]. Parameters were set to maximum e-value <10^−25^, maximum number of alignments to report = 20 and highest scoring pair length = 33 amino acids. BLAST2GO was also used for GO functional enrichment analysis of the transcripts differentially expressed in both germinated spores and haustoria, by performing Fisher’s exact test with false discovery rate (FDR) correction to obtain an adjusted *P*-value. Additionally, BLASTn and BLASTx screening against NCBI non-redundant nucleotide and protein databases were used for manual analysis. Only the transcripts that belonged to one of the functional categories were analysed manually to verify if more than one transcript represented the same gene, in which case it was annotated in Tables S3–S6. For the remainder of the transcripts, which comprise anonymous sequences, this was not possible because different transcripts might have the same top hit without meaning that they were derived from the same gene. The haustorial transcriptome was further analysed with SignalP 3.0 [33] for signal peptide prediction, TMHMM 2.0 [34] for discarding predicted secreted proteins with transmembrane domains and TargetP [35] for discarding predicted secreted proteins with predicted mitochondrial location.

### RT-PCR

Total RNA was isolated from infected (9 dai) and uninfected wheat leaves ground in liquid nitrogen and extracted using QIAGEN (Doncaster Australia) Plant RNeasy kit according to the manufacturer’s instructions. For cDNA preparation, 2 µg of total RNA were mixed with 1 µl of oligo(dT)_18_ and Milli-Q water up to 11 µl and heated at 70°C for 10 min, followed by cooling on ice for one minute. DTT was added to a final concentration of 1 mM together with 1 µl of dNTPs (10 mM), 4 µl of 5× superscript II buffer (supplied with superscript) and 0.5 µl of SuperScript III reverse transcriptase (200 U/µl, Invitrogen). After incubation for 1 h at 42°C, the reaction was stopped by heating 15 min at 70°C and 1 µl of RNase H (1 U/µl) was added to each tube and incubated for 20 min at 37°C. Target cDNAs were amplified using a dilution of 1∶15 of the synthesized cDNA as template, specific primers forward and reverse and 2× PCR Master Mix (Promega). PCR was performed using a MiniCycler (MJ Research) and consisted of 30 cycles of denaturation at 94°C for 30 sec, annealing at 58°C for 30 sec and extension at 72°C for 1 min.

## Supporting Information

Figure S1 **Number of 454 reads per assembled contig for germinated spores and haustorial samples.**
(TIF)

Figure S2 **Length of contigs for germinated spores and haustorial samples assembled from 454 data.**
(TIF)

Figure S3 **Coverage of 454 contigs assembled from 454 data from germinated spores and haustoria samples.**
(TIF)

Figure S4 **Isolated haustoria by affinity chromatography. A.** Stripe rust haustoria isolated by affinity chromatography. Hautoria isolated by ConA affinity chromatography using a 20 µm mesh to filter homogenized tissue and remove plant cell debris. Bright field image with differential-interference contrast, haustoria (black arrows) and contaminating chloroplasts can be seen. **B. Fluorescence microscopy of isolated haustoria by affinity chromatography.** Haustoria were isolated from infected tissue by ConA affinity chromatography as described in Materials and Methods. Bright field and fluorescent images showing isolated haustoria after 30 min incubation with WGA-FITC or ConA-Alexa 594. All images were collected on a Leica DMR epifluorescence microscope.
(TIF)

Table S1 **Transcripts reference set.** 12,282 EST contigs assembled from germinated spores and haustorial datasets were used as references to map Illumina RNA-seq data from the same tissues. Three biological replicates for haustoria (H1, H2, H3) and spores (S1, S2, S3) were used for sequencing. Differences between each tissue were established by RPKM values for each replicate which were subjected to Baggerley’s test to provide statistical support for differences in gene expression.
(XLSX)

Table S2 **Haustorial secreted proteins (HSPs).** A compilation of analyses of the 437 predicted haustorial secreted proteins. The table shows values for expression levels based on Illumina data (RPKM values, green for haustoria and orange for germinated spores), differential expression, cysteine content, BLASTx analysis against the NCBI-nr protein database, BLAST2GO analysis, PFAM motif analysis and Y/F/WxC motif content. In the Y/F/WxC column, 1b30 indicates a single of these motifs before the 30^th^ amino acid, whereas 1a30 represents a single motif after the 30^th^ amino acid.
(XLSX)

Table S3 **General metabolism.** Transcripts encoding proteins with similarities to enzymes involved in metabolic pathways, or with particular roles as described in the text. Expression levels based on Illumina data are presented (RPKM values, green for haustoria and orange for germinated spores) as well as BLASTx analysis against the NCBI-nr protein database.
(XLSX)

Table S4 **Transporters.** Transcripts encoding proteins with similarities to different transporter categories were identified by BLASTx against the NCBI-nr protein database and the Transport Classification Database (TCDB). Expression levels based on Illumina data are presented (RPKM values, green for haustoria and orange for germinated spores).
(XLSX)

Table S5 **Energy metabolism.** Transcripts encoding proteins with similarities to the enzymes of glycolysis, citric acid cycle, and oxidative phosphorylation, and their *in silico* expression levels based on Illumina data are presented (RPKM values, green for haustoria and orange for germinated spores).
(XLSX)

Table S6 **Gene expression values for the ontological analysis presented in** **Figure 4** **.** Transcripts analysed with BLAST2GO and classed into different metabolic categories summarised in Figure 4 are listed in this table. The BLASTx values against the NCBI-nr protein database are included and their *in silico* expression levels based on Illumina data are presented (RPKM values, green for haustoria and orange for germinated spores). Transcripts highlighted in brown were excluded from the analysis due to low RPKM values (<10).
(XLSX)

Table S7 **Illumina sequencing data before and after mapping against the transcripts reference set**. Three biological replicates were sequenced with Illumina for isolated haustoria (H) and germinated spores (S). The table shows the millions of reads obtained per replicate and the percentage of reads mapping against the transcripts reference set assembled from 454 haustoria and germinated spore data.
(DOCX)

Table S8 **List of primers to validate candidate effector gene expression by non-quantitative RT-PCR.**
(XLSX)

Data S1 **Germinated spores transcriptome.**
(ZIP)

Data S2 **Haustoria transcriptome.**
(ZIP)

Data S3 **437 HSP ORFs.**
(ZIP)

Data S4 **PST79 combined transcriptome.**
(ZIP)

Text S1 **Supplementary text.**
(DOCX)

## References

[1] Wellings CR (2007) *Puccinia striiformis* in Australia: a review of the incursion, evolution, and adaptation of stripe rust in the period 1979–2006. Australian Journal of Agricultural Research 58: 567–575.

[2] Murray G, Brennan JP (2009) The Current and Potential Costs from Diseases of Wheat in Australia. GRDC Available: http://wwwgrdccomau/uploads/documents/GRDC_WheatDiseaseLoss_Report_finalpdf Accessed 2012 Jan 20.

[3] Wellings C (2011) Global status of stripe rust: a review of historical and current threats. Euphytica 179: 129–141.

[4] Wan AM, Zhao ZH, Chen XM, He ZH, Jin SL, et al (2004) Wheat stripe rust epidemic and virulence of *Puccinia striiformis* f. sp *tritici* in China in 2002. Plant Disease 88: 896–904. 10.1094/PDIS.2004.88.8.896 30812521

[5] Chen XM (2007) Challenges and solutions for stripe rust control in the United States. Australian Journal of Agricultural Research 58: 648–655.

[6] Marasas CN, Smale M, Singh RP (2004) The economic impact in developing countries of leaf rust resistance breeding in CIMMYT-related spring bread wheat. CIMMYT Available: http://impactcgiarorg/pdf/274pdf Accessed 30 January 2012.

[7] Kolmer J, Ordonez M, Groth J (2009) The Rust Fungi. In: John Wiley & Sons L, editor. Encyclopedia of Life Sciences (ELS). Chichester.

[8] Jin Y, Szabo LJ, Carson M (2010) Century-Old Mystery of *Puccinia striiformis* Life History Solved with the Identification of Berberis as an Alternate Host. Phytopathology 100: 432–435. 20373963 10.1094/PHYTO-100-5-0432

[9] Fabro G, Di Rienzo JA, Voigt CA, Savchenko T, Dehesh K, et al (2008) Genome-wide expression profiling *Arabidopsis* at the stage of *Golovinomyces cichoracearum* haustorium formation. Plant Physiology 146: 1421–1439. 18218973 10.1104/pp.107.111286 PMC2259087

[10] Horst RJ, Doehlemann G, Wahl R, Hofmann J, Schmiedl A, et al (2010) Ustilago maydis infection strongly alters organic nitrogen allocation in maize and stimulates productivity of systemic source leaves. Plant Physiol 152: 293–308. 19923237 10.1104/pp.109.147702 PMC2799364

[11] Spanu P, Kamper J (2010) Genomics of biotrophy in fungi and oomycetes-emerging patterns. Current Opinion in Plant Biology 13: 409–414. 20430688 10.1016/j.pbi.2010.03.004

[12] Szabo LJ, Bushnell WR (2001) Hidden robbers: The role of fungal haustoria in parasitism of plants. Proc Natl Acad Sci U S A 98: 7654–7655. 11438718 10.1073/pnas.151262398 PMC35395

[13] Hahn M, Mendgen K (1997) Characterization of in planta induced rust genes isolated from a haustorium-specific cDNA library. Molecular Plant-Microbe Interactions 10: 427–437. 9150592 10.1094/MPMI.1997.10.4.427

[14] Voegele RT, Struck C, Hahn M, Mendgen K (2001) The role of haustoria in sugar supply during infection of broad bean by the rust fungus Uromyces fabae. Proc Natl Acad Sci U S A 98: 8133–8138. 11390980 10.1073/pnas.131186798 PMC35480

[15] Heath MC (1997) Signalling between pathogenic rust fungi and resistant or susceptible host plants. Annals of Botany 80: 713–720.

[16] Voegele RT, Mendgen K (2003) Rust haustoria: nutrient uptake and beyond. New Phytologist 159: 93–100. 10.1046/j.1469-8137.2003.00761.x 33873671

[17] Stergiopoulos I, de Wit PJGM (2009) Fungal Effector Proteins. Annual Review of Phytopathology 47: 233–263. 10.1146/annurev.phyto.112408.132637 19400631

[18] Kemen E, Kemen AC, Rafiqi M, Hempel U, Mendgen K, et al (2005) Identification of a protein from rust fungi transferred from haustoria into infected plant cells. Molecular Plant-Microbe Interactions 18: 1130–1139. 16353548 10.1094/MPMI-18-1130

[19] Rafiqi M, Gan P, Ravensdale M, Lawrence G, Ellis J, et al (2010) Internalization of Flax Rust Avirulence Proteins into Flax and Tobacco Cells Can Occur in the Absence of the Pathogen. The Plant Cell 22: 2017–2032. 20525849 10.1105/tpc.109.072983 PMC2910983

[20] Panstruga R, Dodds PN (2009) Terrific protein traffic: the mystery of effector protein delivery by filamentous plant pathogens. Science 324: 748–750. 19423815 10.1126/science.1171652 PMC2775090

[21] Bent AF, Mackey D (2007) Elicitors, effectors, and R genes: the new paradigm and a lifetime supply of questions. Annual Review of Phytopathology 45: 399–436. 10.1146/annurev.phyto.45.062806.094427 17506648

[22] Hahn M, Neef U, Struck C, Gottfert M, Mendgen K (1997) A putative amino acid transporter is specifically expressed in haustoria of the rust fungus *Uromyces fabae* . Molecular Plant-Microbe Interactions 10: 438–445. 9150593 10.1094/MPMI.1997.10.4.438

[23] Duplessis S, Cuomo CA, Lin YC, Aerts A, Tisserant E, et al (2011) Obligate biotrophy features unraveled by the genomic analysis of rust fungi. Proc Natl Acad Sci U S A 108: 9166–9171. 21536894 10.1073/pnas.1019315108 PMC3107277

[24] Spanu PD, Abbott JC, Amselem J, Burgis TA, Soanes DM, et al (2010) Genome expansion and gene loss in powdery mildew fungi reveal tradeoffs in extreme parasitism. Science 330: 1543–1546. 21148392 10.1126/science.1194573

[25] Ling P, Wang M, Chen X, Campbell KG (2007) Construction and characterization of a full-length cDNA library for the wheat stripe rust pathogen (Puccinia striiformis f. sp. tritici). BMC Genomics 8: 145. 17547766 10.1186/1471-2164-8-145 PMC1903366

[26] Ma J, Huang X, Wang X, Chen X, Qu Z, et al (2009) Identification of expressed genes during compatible interaction between stripe rust (*Puccinia striiformis*) and wheat using a cDNA library. BMC Genomics 10: 586. 19995415 10.1186/1471-2164-10-586 PMC3087560

[27] Zhang Y, Qu Z, Zheng W, Liu B, Wang X, et al (2008) Stage-specific gene expression during urediniospore germination in *Puccinia striiformis* f. sp *tritici* . BMC Genomics 9: 203. 18447959 10.1186/1471-2164-9-203 PMC2386484

[28] Thara VK, Fellers JP, Zhou JM (2003) In planta induced genes of *Puccinia triticina* . Molecular Plant Pathology 4: 51–56. 20569362 10.1046/j.1364-3703.2003.00142.x

[29] Hu GG, Linning R, Mccallum B, Banks T, Cloutier S, et al (2007) Generation of a wheat leaf rust, *Puccinia triticina*, EST database from stage-specific cDNA libraries. Molecular Plant Pathology 8: 451–467. 20507513 10.1111/j.1364-3703.2007.00406.x

[30] Broeker K, Bernard F, Moerschbacher BM (2006) An EST library from *Puccinia graminis* f. sp. *tritici* reveals genes potentially involved in fungal differentiation. FEMS Microbiol Lett 256: 273–281. 16499617 10.1111/j.1574-6968.2006.00127.x

[31] Hahn M, Mendgen K (1992) Isolation by Cona Binding of Haustoria from Different Rust Fungi and Comparison of Their Surface Qualities. Protoplasma 170: 95–103.

[32] Cantu D, Govindarajulu M, Kozik A, Wang M, Chen X, et al (2011) Next generation sequencing provides rapid access to the genome of *Puccinia striiformis* f. sp. *tritici*, the causal agent of wheat stripe rust. PLoS One 6: e24230. 21909385 10.1371/journal.pone.0024230 PMC3164196

[33] Bendtsen JD, Nielsen H, von Heijne G, Brunak S (2004) Improved prediction of signal peptides: SignalP 3.0. Journal of Molecular Biology 340: 783–795. 15223320 10.1016/j.jmb.2004.05.028

[34] Krogh A, Larsson B, von Heijne G, Sonnhammer EL (2001) Predicting transmembrane protein topology with a hidden Markov model: application to complete genomes. Journal of Molecular Biology 305: 567–580. 11152613 10.1006/jmbi.2000.4315

[35] Emanuelsson O, Brunak S, von Heijne G, Nielsen H (2007) Locating proteins in the cell using TargetP, SignalP and related tools. Nature Protocols 2: 953–971. 17446895 10.1038/nprot.2007.131

[36] Conesa A, Gotz S, Garcia-Gomez JM, Terol J, Talon M, et al (2005) Blast2GO: a universal tool for annotation, visualization and analysis in functional genomics research. Bioinformatics 21: 3674–3676. 16081474 10.1093/bioinformatics/bti610

[37] Dong YL, Yin CT, Hulbert S, Chen XM, Kang ZS (2011) Cloning and expression analysis of three secreted protein genes from wheat stripe rust fungus *Puccinia striiformis f. sp. tritici* . World Journal of Microbiology and Biotechnology 27: 1261–1265.

[38] Saunders DGO, Win J, Cano LM, Szabo LJ, Kamoun S, et al (2012) Using Hierarchical Clustering of Secreted Protein Families to Classify and Rank Candidate Effectors of Rust Fungi. PLoS One 7: e29847. 22238666 10.1371/journal.pone.0029847 PMC3253089

[39] Bhadauria V, Banniza S, Vandenberg A, Selvaraj G, Wei Y (2012) Overexpression of a Novel Biotrophy-Specific *Colletotrichum truncatum* Effector CtNUDIX in Hemibiotrophic Fungal Phytopathogens Causes Incompatibility with Their Host Plants. Eukaryotic Cell 12: 2–11. 22962277 10.1128/EC.00192-12 PMC3535838

[40] Hacquard S, Joly DL, Lin YC, Tisserant E, Feau N, et al (2012) A comprehensive analysis of genes encoding small secreted proteins identifies candidate effectors in Melampsora larici-populina (poplar leaf rust). Molecular Plant-Microbe Interactions 25: 279–293. 22046958 10.1094/MPMI-09-11-0238

[41] Mortazavi A, Williams BA, McCue K, Schaeffer L, Wold B (2008) Mapping and quantifying mammalian transcriptomes by RNA-Seq. Nat Methods 5: 621–628. 18516045 10.1038/nmeth.1226 PMC13303166

[42] Baggerly KA, Deng L, Morris JS, Aldaz CM (2003) Differential expression in SAGE: accounting for normal between-library variation. Bioinformatics 19: 1477–1483. 12912827 10.1093/bioinformatics/btg173

[43] Flor-Parra I, Castillo-Lluva S, Perez-Martin J (2007) Polar growth in the infectious hyphae of the phytopathogen *Ustilago maydis* depends on a virulence-specific cyclin. Plant Cell 19: 3280–3296. 17921314 10.1105/tpc.107.052738 PMC2174706

[44] Garcia-Muse T, Steinberg G, Perez-Martin J (2004) Characterization of B-type cyclins in the smut fungus *Ustilago maydis*: roles in morphogenesis and pathogenicity. J Cell Sci 117: 487–506. 14679309 10.1242/jcs.00877

[45] Saunders DG, Aves SJ, Talbot NJ (2010) Cell cycle-mediated regulation of plant infection by the rice blast fungus. Plant Cell 22: 497–507. 20190078 10.1105/tpc.109.072447 PMC2845407

[46] Cid VJ, Adamikova L, Sanchez M, Molina M, Nombela C (2001) Cell cycle control of septin ring dynamics in the budding yeast. Microbiology 147: 1437–1450. 11390675 10.1099/00221287-147-6-1437

[47] Dagdas YF, Yoshino K, Dagdas G, Ryder LS, Bielska E, et al (2012) Septin-mediated plant cell invasion by the rice blast fungus, *Magnaporthe oryzae* . Science 336: 1590–1595. 22723425 10.1126/science.1222934

[48] Carpita NC, Gibeaut DM (1993) Structural models of primary cell walls in flowering plants: consistency of molecular structure with the physical properties of the walls during growth. Plant Journal 3: 1–30. 8401598 10.1111/j.1365-313x.1993.tb00007.x

[49] Bowman SM, Free SJ (2006) The structure and synthesis of the fungal cell wall. Bioessays 28: 799–808. 16927300 10.1002/bies.20441

[50] Soanes DM, Richards TA, Talbot NJ (2007) Insights from sequencing fungal and oomycete genomes: what can we learn about plant disease and the evolution of pathogenicity? Plant Cell 19: 3318–3326. 18024565 10.1105/tpc.107.056663 PMC2174898

[51] Ipcho SV, Hane JK, Antoni EA, Ahren D, Henrissat B, et al (2012) Transcriptome analysis of *Stagonospora nodorum*: gene models, effectors, metabolism and pantothenate dispensability. Mol Plant Pathol 13: 531–545. 22145589 10.1111/j.1364-3703.2011.00770.x PMC6638697

[52] Dean RA, Talbot NJ, Ebbole DJ, Farman ML, Mitchell TK, et al (2005) The genome sequence of the rice blast fungus *Magnaporthe grisea* . Nature 434: 980–986. 15846337 10.1038/nature03449

[53] Kamper J, Kahmann R, Bolker M, Ma LJ, Brefort T, et al (2006) Insights from the genome of the biotrophic fungal plant pathogen Ustilago maydis. Nature 444: 97–101. 17080091 10.1038/nature05248

[54] Chrost B, Kolukisaoglu U, Schulz B, Krupinska K (2007) An alpha-galactosidase with an essential function during leaf development. Planta 225: 311–320. 16845526 10.1007/s00425-006-0350-9

[55] Gueddari NE, Rauchhaus U, Moerschbacher BM, Deising HB (2002) Developmentally regulated conversion of surface-exposed chitin to chitosan in cell walls of plant pathogenic fungi. New Physiologist: 103–112.

[56] Vincent D, Kohler A, Claverol S, Solier E, Joets J, et al (2012) Secretome of the free-living mycelium from the ectomycorrhizal basidiomycete *Laccaria bicolor* . Journal of Proteome Research 11: 157–171. 22074047 10.1021/pr200895f

[57] Vieira A, Talhinhas P, Loureiro A, Thurich J, Duplessis S, et al (2012) Expression profiling of genes involved in the biotrophic colonisation of *Coffea arabica* leaves by *Hemileia vastatrix* . European Journal of Plant Pathology 133: 261–277.

[58] Deising H, Siegrist J (1995) Chitin Deacetylase Activity of the Rust *Uromyces viciae-fabae* Is Controlled by Fungal Morphogenesis. Fems Microbiology Letters 127: 207–211.

[59] Struck C, Ernst M, Hahn M (2002) Characterization of a developmentally regulated amino acid transporter (AAT1p) of the rust fungus *Uromyces fabae* . Mol Plant Pathol 3: 23–30. 20569305 10.1046/j.1464-6722.2001.00091.x

[60] Struck C, Mueller E, Martin H, Lohaus G (2004) The *Uromyces fabae* UfAAT3 gene encodes a general amino acid permease that prefers uptake of in planta scarce amino acids. Mol Plant Pathol 5: 183–189. 20565608 10.1111/j.1364-3703.2004.00222.x

[61] Rees DC, Johnson E, Lewinson O (2009) ABC transporters: the power to change. Nat Rev Mol Cell Biol 10: 218–227. 19234479 10.1038/nrm2646 PMC2830722

[62] Pao SS, Paulsen IT, Saier MH Jr (1998) Major facilitator superfamily. Microbiol Mol Biol Rev 62: 1–34. 9529885 10.1128/mmbr.62.1.1-34.1998 PMC98904

[63] Sun CB, Suresh A, Deng YZ, Naqvi NI (2006) A multidrug resistance transporter in Magnaporthe is required for host penetration and for survival during oxidative stress. Plant Cell 18: 3686–3705. 17189344 10.1105/tpc.105.037861 PMC1785395

[64] Gupta A, Chattoo BB (2008) Functional analysis of a novel ABC transporter ABC4 from *Magnaporthe grisea* . FEMS Microbiol Lett 278: 22–28. 18034832 10.1111/j.1574-6968.2007.00937.x

[65] Soanes DM, Chakrabarti A, Paszkiewicz KH, Dawe AL, Talbot NJ (2012) Genome-wide transcriptional profiling of appressorium development by the rice blast fungus *Magnaporthe oryzae* . PLoS Pathog 8: e1002514. 22346750 10.1371/journal.ppat.1002514 PMC3276559

[66] Voegele R, Mendgen KW (2011) Nutrient uptake in rust fungi: how sweet is parasitic life? Euphytica 179: 41–55.

[67] Struck C, Siebels C, Rommel O, Wernitz M, Hahn M (1998) The plasma membrane H(+)-ATPase from the biotrophic rust fungus *Uromyces fabae*: molecular characterization of the gene (PMA1) and functional expression of the enzyme in yeast. Mol Plant Microbe Interact 11: 458–465. 9612944 10.1094/MPMI.1998.11.6.458

[68] Struck C, Hahn M, Mendgen K (1996) Plasma Membrane H+-ATPase Activity in Spores, Germ Tubes, and Haustoria of the Rust Fungus *Uromyces viciae-fabae* . Fungal Genet Biol 20: 30–35. 8812284 10.1006/fgbi.1996.0006

[69] Ferreira C, van Voorst F, Martins A, Neves L, Oliveira R, et al (2005) A member of the sugar transporter family, Stl1p is the glycerol/H+ symporter in *Saccharomyces cerevisiae* . Mol Biol Cell 16: 2068–2076. 15703210 10.1091/mbc.E04-10-0884 PMC1073684

[70] Ansell R, Granath K, Hohmann S, Thevelein JM, Adler L (1997) The two isoenzymes for yeast NAD+-dependent glycerol 3-phosphate dehydrogenase encoded by GPD1 and GPD2 have distinct roles in osmoadaptation and redox regulation. EMBO J 16: 2179–2187. 9171333 10.1093/emboj/16.9.2179 PMC1169820

[71] Castro IM, Loureiro-Dias MC (1991) Glycerol utilization in *Fusarium oxysporum* var. *lini*: regulation of transport and metabolism. J Gen Microbiol 137: 1497–1502. 1955848 10.1099/00221287-137-7-1497

[72] Wei Y, Shen W, Dauk M, Wang F, Selvaraj G, et al (2004) Targeted gene disruption of glycerol-3-phosphate dehydrogenase in *Colletotrichum gloeosporioides* reveals evidence that glycerol is a significant transferred nutrient from host plant to fungal pathogen. J Biol Chem 279: 429–435. 14563847 10.1074/jbc.M308363200

[73] Scarpari LM, Meinhardt LW, Mazzafera P, Pomella AW, Schiavinato MA, et al (2005) Biochemical changes during the development of witches’ broom: the most important disease of cocoa in Brazil caused by *Crinipellis perniciosa* . J Exp Bot 56: 865–877. 15642708 10.1093/jxb/eri079

[74] Wykoff DD, O’Shea EK (2001) Phosphate transport and sensing in *Saccharomyces cerevisiae* . Genetics 159: 1491–1499. 11779791 10.1093/genetics/159.4.1491 PMC1450841

[75] Bun-Ya M, Nishimura M, Harashima S, Oshima Y (1991) The PHO84 gene of *Saccharomyces cerevisiae* encodes an inorganic phosphate transporter. Mol Cell Biol 11: 3229–3238. 2038328 10.1128/mcb.11.6.3229-3238.1991 PMC360175

[76] Secco D, Wang C, Arpat BA, Wang Z, Poirier Y, et al (2012) The emerging importance of the SPX domain-containing proteins in phosphate homeostasis. New Phytol 193: 842–851. 22403821 10.1111/j.1469-8137.2011.04002.x

[77] Nieuwenhuis BJ, Borst-Pauwels GW (1984) Derepression of the high-affinity phosphate uptake in the yeast *Saccharomyces cerevisiae* . Biochim Biophys Acta 770: 40–46. 6365165 10.1016/0005-2736(84)90071-3

[78] Lenburg ME, O’Shea EK (1996) Signaling phosphate starvation. Trends Biochem Sci 21: 383–387. 8918192

[79] Popova Y, Thayumanavan P, Lonati E, Agrochao M, Thevelein JM (2010) Transport and signaling through the phosphate-binding site of the yeast Pho84 phosphate transceptor. Proc Natl Acad Sci U S A 107: 2890–2895. 20133652 10.1073/pnas.0906546107 PMC2840322

[80] Llorente B, Dujon B (2000) Transcriptional regulation of the *Saccharomyces cerevisiae* DAL5 gene family and identification of the high affinity nicotinic acid permease TNA1 (YGR260w). FEBS Lett 475: 237–241. 10869563 10.1016/s0014-5793(00)01698-7

[81] Jakupovic M, Heintz M, Reichmann P, Mendgen K, Hahn M (2006) Microarray analysis of expressed sequence tags from haustoria of the rust fungus *Uromyces fabae* . Fungal Genet Biol 43: 8–19. 16289953 10.1016/j.fgb.2005.09.001

[82] Both M, Csukai M, Stumpf MP, Spanu PD (2005) Gene expression profiles of *Blumeria graminis* indicate dynamic changes to primary metabolism during development of an obligate biotrophic pathogen. Plant Cell 17: 2107–2122. 15951491 10.1105/tpc.105.032631 PMC1167555

[83] Ehrlich MA, Ehrlich HG (1969) Uredinospore development in *Puccinia graminis* . Canadian Journal of Botany 47: 2061–2064.

[84] Thines E, Weber RW, Talbot NJ (2000) MAP kinase and protein kinase A-dependent mobilization of triacylglycerol and glycogen during appressorium turgor generation by *Magnaporthe grisea* . Plant Cell 12: 1703–1718. 11006342 10.1105/tpc.12.9.1703 PMC149080

[85] Bozdech Z, Ginsburg H (2005) Data mining of the transcriptome of *Plasmodium falciparum*: the pentose phosphate pathway and ancillary processes. Malar J 4: 17. 15774020 10.1186/1475-2875-4-17 PMC1084361

[86] Bozdech Z, Llinas M, Pulliam BL, Wong ED, Zhu J, et al (2003) The transcriptome of the intraerythrocytic developmental cycle of *Plasmodium falciparum* . PLoS Biol 1: E5. 12929205 10.1371/journal.pbio.0000005 PMC176545

[87] Kemen E, Gardiner A, Schultz-Larsen T, Kemen AC, Balmuth AL, et al (2011) Gene gain and loss during evolution of obligate parasitism in the white rust pathogen of *Arabidopsis thaliana* . PLoS Biol 9: e1001094. 21750662 10.1371/journal.pbio.1001094 PMC3130010

[88] Meti RS, Ambarish S, Khajure PV (2011) Enzymes of Ammonia Assimilation in Fungi: An Overview. Recent Research in Science and Technology 2: 28–38.

[89] Marzluf GA (1997) Molecular genetics of sulfur assimilation in filamentous fungi and yeast. Annu Rev Microbiol 51: 73–96. 9343344 10.1146/annurev.micro.51.1.73

[90] Baxter L, Tripathy S, Ishaque N, Boot N, Cabral A, et al (2010) Signatures of adaptation to obligate biotrophy in the *Hyaloperonospora arabidopsidis* genome. Science 330: 1549–1551. 21148394 10.1126/science.1195203 PMC3971456

[91] Bourgis F, Roje S, Nuccio ML, Fisher DB, Tarczynski MC, et al (1999) S-methylmethionine plays a major role in phloem sulfur transport and is synthesized by a novel type of methyltransferase. Plant Cell 11: 1485–1498. 10449582 10.1105/tpc.11.8.1485 PMC144290

[92] Pocsi I, Prade RA, Penninckx MJ (2004) Glutathione, altruistic metabolite in fungi. Adv Microb Physiol 49: 1–76. 15518828 10.1016/S0065-2911(04)49001-8

[93] Deng J, Carbone I, Dean RA (2007) The evolutionary history of cytochrome P450 genes in four filamentous Ascomycetes. BMC Evol Biol 7: 30. 17324274 10.1186/1471-2148-7-30 PMC1828051

[94] Vanetten HD, Sandrock RW, Wasmann CC, Soby SD, Mccluskey K, et al (1995) Detoxification of Phytoanticipins and Phytoalexins by Phytopathogenic Fungi. Canadian Journal of Botany 73: S518–S525.

[95] Vanden Bossche H, Marichal P, Gorrens J, Coene MC (1990) Biochemical basis for the activity and selectivity of oral antifungal drugs. Br J Clin Pract Suppl 71: 41–46. 2091733

[96] Wong Sak Hoi J, Dumas B (2010) Ste12 and Ste12-like proteins, fungal transcription factors regulating development and pathogenicity. Eukaryot Cell 9: 480–485. 20139240 10.1128/EC.00333-09 PMC2863410

[97] Madhani HD, Fink GR (1997) Combinatorial control required for the specificity of yeast MAPK signaling. Science 275: 1314–1317. 9036858 10.1126/science.275.5304.1314

[98] Liu H, Kohler J, Fink GR (1994) Suppression of hyphal formation in Candida albicans by mutation of a STE12 homolog. Science 266: 1723–1726. 7992058 10.1126/science.7992058

[99] Calcagno AM, Bignell E, Warn P, Jones MD, Denning DW, et al (2003) *Candida glabrata* STE12 is required for wild-type levels of virulence and nitrogen starvation induced filamentation. Molecular Microbiology 50: 1309–1318. 14622417 10.1046/j.1365-2958.2003.03755.x

[100] Tsuji G, Sugahara T, Fujii I, Mori Y, Ebizuka Y, et al (2003) Evidence for involvement of two naphthol reductases in the first reduction step of melanin biosynthesis pathway of *Colletotrichum lagenarium* . Mycol Res 107: 854–860. 12967213 10.1017/s0953756203008001

[101] Chang YC, Penoyer LA, Kwon-Chung KJ (2001) The second STE12 homologue of *Cryptococcus neoformans* is MATa-specific and plays an important role in virulence. Proc Natl Acad Sci U S A 98: 3258–3263. 11248066 10.1073/pnas.061031998 PMC30641

[102] Rispail N, Di Pietro A (2009) *Fusarium oxysporum* Ste12 controls invasive growth and virulence downstream of the Fmk1 MAPK cascade. Mol Plant Microbe Interact 22: 830–839. 19522565 10.1094/MPMI-22-7-0830

[103] Park G, Xue C, Zheng L, Lam S, Xu JR (2002) MST12 regulates infectious growth but not appressorium formation in the rice blast fungus *Magnaporthe grisea* . Mol Plant Microbe Interact 15: 183–192. 11952120 10.1094/MPMI.2002.15.3.183

[104] Wong Sak Hoi J, Herbert C, Bacha N, O’Connell R, Lafitte C, et al (2007) Regulation and role of a STE12-like transcription factor from the plant pathogen *Colletotrichum lindemuthianum* . Molecular Microbiology 64: 68–82. 17376073 10.1111/j.1365-2958.2007.05639.x

[105] Tollot M, Wong Sak Hoi J, van Tuinen D, Arnould C, Chatagnier O, et al (2009) An STE12 gene identified in the mycorrhizal fungus Glomus intraradices restores infectivity of a hemibiotrophic plant pathogen. New Phytol 181: 693–707. 19140944 10.1111/j.1469-8137.2008.02696.x

[106] Nadal M, Garcia-Pedrajas MD, Gold SE (2008) Dimorphism in fungal plant pathogens. FEMS Microbiol Lett 284: 127–134. 18479435 10.1111/j.1574-6968.2008.01173.x

[107] Grbavec D, Lo R, Liu Y, Greenfield A, Stifani S (1999) Groucho/transducin-like enhancer of split (TLE) family members interact with the yeast transcriptional co-repressor SSN6 and mammalian SSN6-related proteins: implications for evolutionary conservation of transcription repression mechanisms. Biochem J 337 (Pt 1): 13–17. PMC1219929 9854018

[108] Elias-Villalobos A, Fernandez-Alvarez A, Ibeas JI (2011) The general transcriptional repressor Tup1 is required for dimorphism and virulence in a fungal plant pathogen. PLoS Pathog 7: e1002235. 21909277 10.1371/journal.ppat.1002235 PMC3164652

[109] Paietta JV, Akins RA, Lambowitz AM, Marzluf GA (1987) Molecular cloning and characterization of the cys-3 regulatory gene of *Neurospora crassa* . Mol Cell Biol 7: 2506–2511. 2886908 10.1128/mcb.7.7.2506-2511.1987 PMC365384

[110] Tao Y, Marzluf GA (1998) Synthesis and differential turnover of the CYS3 regulatory protein of *Neurospora crassa* are subject to sulfur control. J Bacteriol 180: 478–482. 9457847 10.1128/jb.180.3.478-482.1998 PMC106911

[111] Bahn YS (2008) Master and commander in fungal pathogens: the two-component system and the HOG signaling pathway. Eukaryot Cell 7: 2017–2036. 18952900 10.1128/EC.00323-08 PMC2593196

[112] Oide S, Liu J, Yun SH, Wu D, Michev A, et al (2010) Histidine kinase two-component response regulator proteins regulate reproductive development, virulence, and stress responses of the fungal cereal pathogens *Cochliobolus heterostrophus* and *Gibberella zeae* . Eukaryot Cell 9: 1867–1880. 21037181 10.1128/EC.00150-10 PMC3008274

[113] Catlett NL, Yoder OC, Turgeon BG (2003) Whole-genome analysis of two-component signal transduction genes in fungal pathogens. Eukaryot Cell 2: 1151–1161. 14665450 10.1128/EC.2.6.1151-1161.2003 PMC326637

[114] Alex LA, Borkovich KA, Simon MI (1996) Hyphal development in *Neurospora crassa*: involvement of a two-component histidine kinase. Proc Natl Acad Sci U S A 93: 3416–3421. 8622950 10.1073/pnas.93.8.3416 PMC39623

[115] Schumacher MM, Enderlin CS, Selitrennikoff CP (1997) The osmotic-1 locus of *Neurospora crassa* encodes a putative histidine kinase similar to osmosensors of bacteria and yeast. Curr Microbiol 34: 340–347. 9142740 10.1007/s002849900193

[116] Cui W, Beever RE, Parkes SL, Weeds PL, Templeton MD (2002) An osmosensing histidine kinase mediates dicarboximide fungicide resistance in *Botryotinia fuckeliana* (*Botrytis cinerea*). Fungal Genet Biol 36: 187–198. 12135574 10.1016/s1087-1845(02)00009-9

[117] Pillonel C, Meyer T (1997) Effect of phenylpyrroles on glycerol accumulation and protein kinase activity of *Neurospora crassa* . Pesticide Science 49: 229–236.

[118] Howard RJ, Valent B (1996) Breaking and entering: host penetration by the fungal rice blast pathogen *Magnaporthe grisea* . Annu Rev Microbiol 50: 491–512. 8905089 10.1146/annurev.micro.50.1.491

[119] Motoyama T, Kadokura K, Ohira T, Ichiishi A, Fujimura M, et al (2005) A two-component histidine kinase of the rice blast fungus is involved in osmotic stress response and fungicide action. Fungal Genetics and Biology 42: 200–212. 15707841 10.1016/j.fgb.2004.11.002

[120] Staples RC, Wynn WK (1965) The Physiology of Uredospores of the Rust Fungi. Botanical Review 31: 537–564.

[121] Harder D (1984) Developmental ultrastructure of hyphae and spores. In: Bushnell W, Roelfs A, editors. The cereal Rust Vol I. St. Paul, Minnesota: Cereal Rust Laboratory, U.S. Department of Agriculture: Academic Press, Inc.

[122] Hahn M, Deising H, Struck C, Mendgen K (1997) Fungal morphogenesis and enzyme secretion during pathogenesis. In: Hartleb H, Heitefuss R, Hoppe H, editors. Resistance of crop plants against fungi Lübeck, Ulm: Gustav Fischer. 33–57.

[123] Catanzariti AM, Mago R, Ellis J, Dodds P (2011) Constructing haustorium-specific cDNA libraries from rust fungi. Methods Mol Biol 712: 79–87. 21359802 10.1007/978-1-61737-998-7_8

