# Supplementary figures and images for: Strategies for Wheat Stripe Rust Pathogenicity Identified by Transcriptome Sequencing
Source: PLoS One. 2013 Jun 26;8(6):e67150. doi: 10.1371/journal.pone.0067150 (PMC3694141; doi:10.1371/journal.pone.0067150)

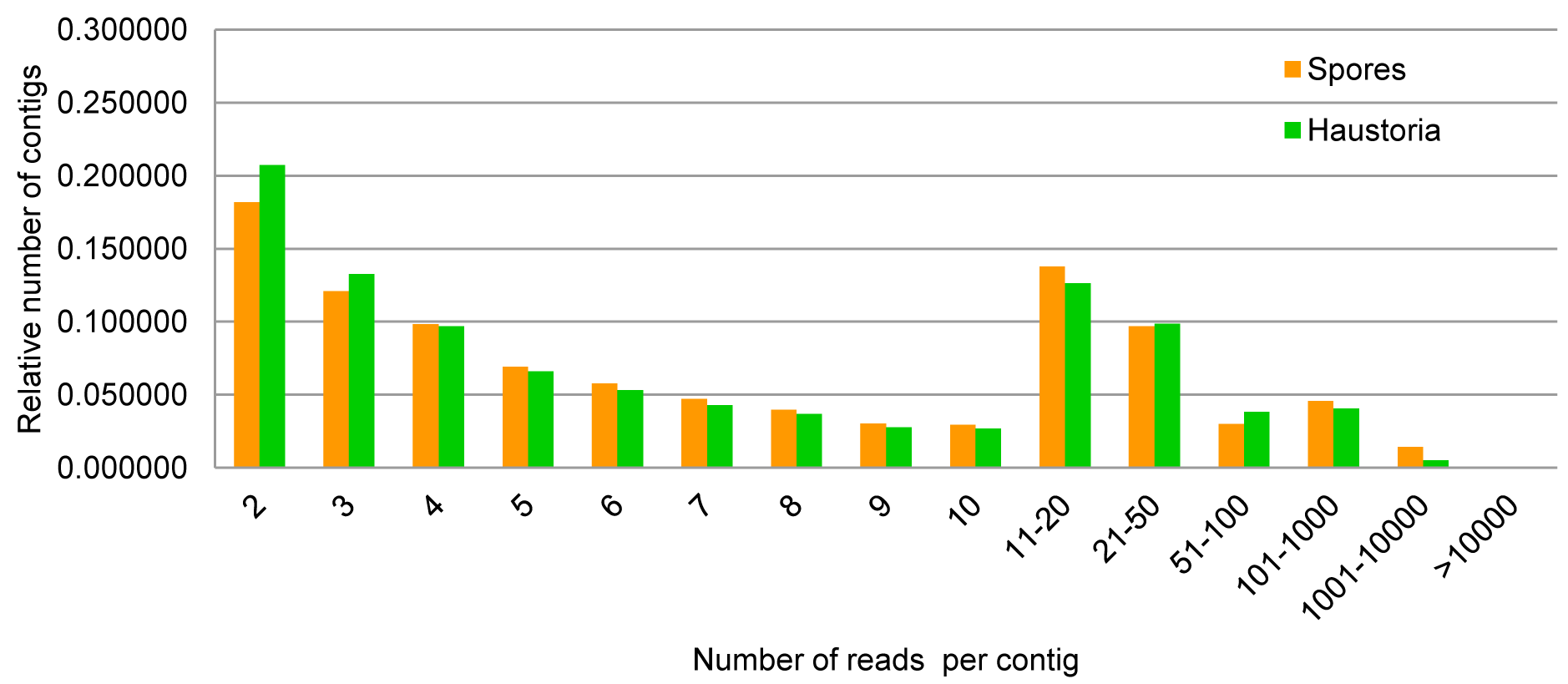

Supplement: Figure S1 — Number of 454 reads per assembled contig for germinated spores and haustorial samples. (TIF) [file pone.0067150.s001.tif]

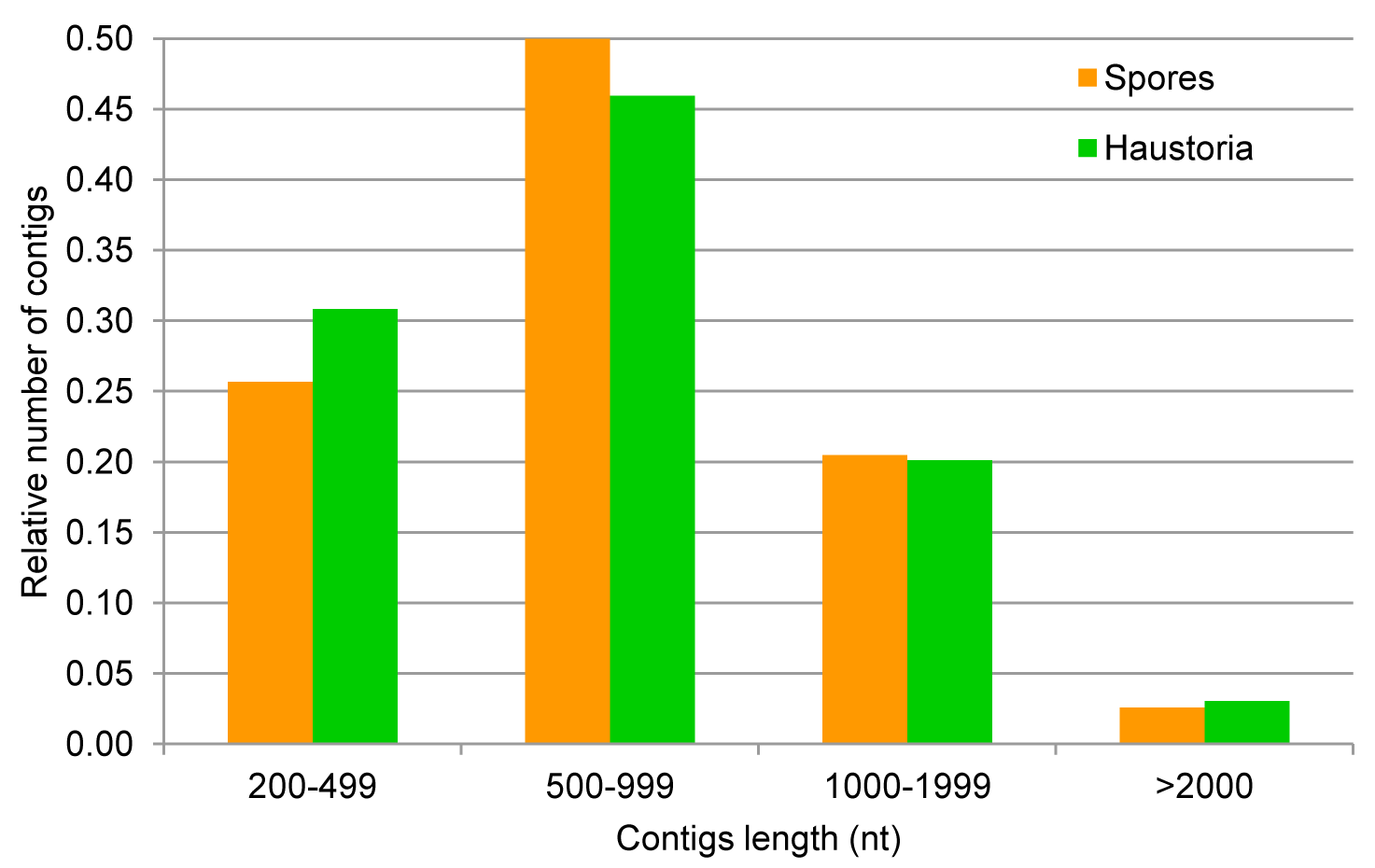

Supplement: Figure S2 — Length of contigs for germinated spores and haustorial samples assembled from 454 data. (TIF) [file pone.0067150.s002.tif]

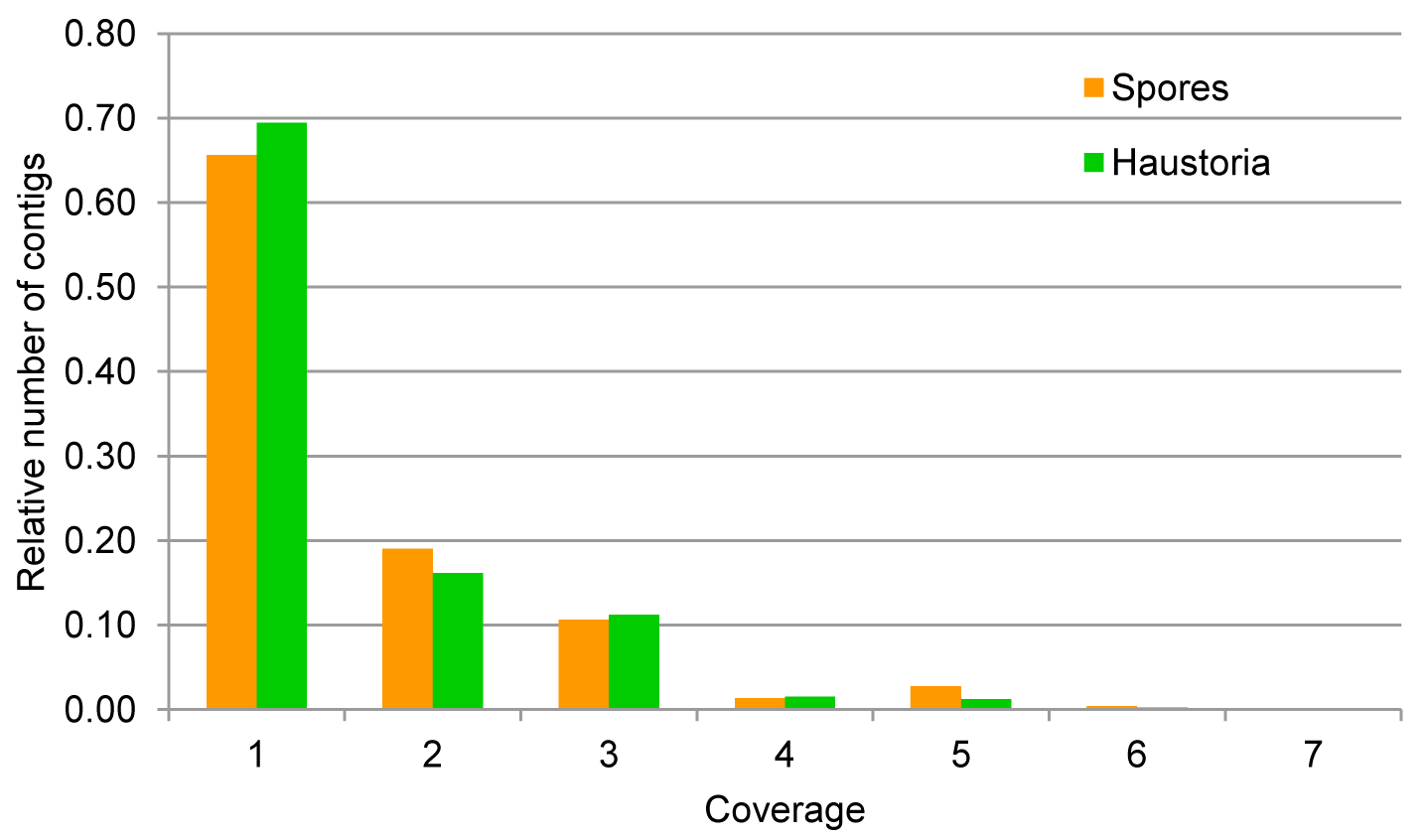

Supplement: Figure S3 — Coverage of 454 contigs assembled from 454 data from germinated spores and haustoria samples. (TIF) [file pone.0067150.s003.tif]

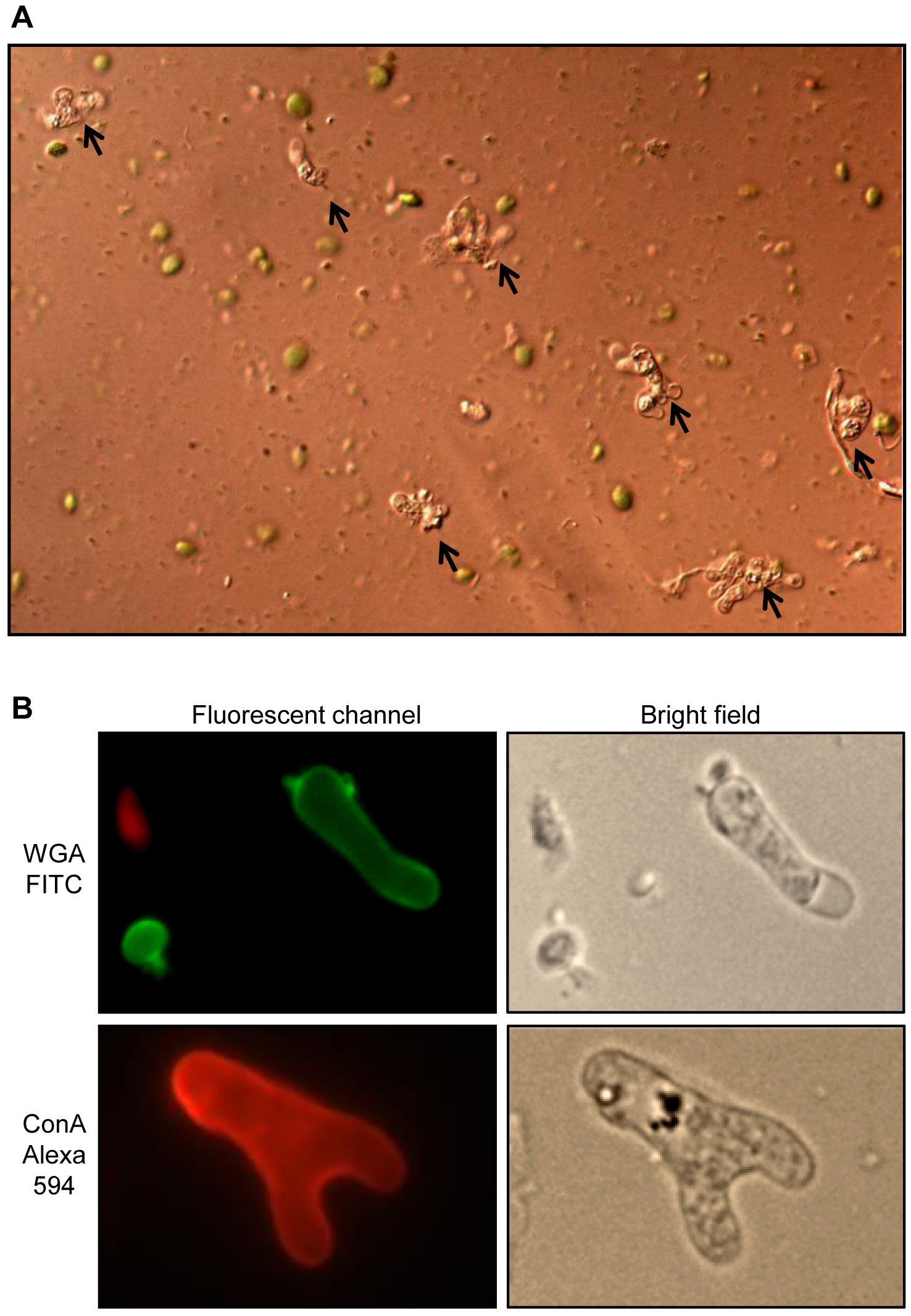

Supplement: Figure S4 — Isolated haustoria by affinity chromatography. A. Stripe rust haustoria isolated by affinity chromatography. Hautoria isolated by ConA affinity chromatography using a 20 µm mesh to filter homogenized tissue and remove plant cell debris. Bright field image with differential-interference contrast, haustoria (black arrows) and contaminating chloroplasts can be seen. B. Fluorescence microscopy of isolated haustoria by affinity chromatography. Haustoria were isolated from infected tissue by ConA affinity chromatography as described in Materials and Methods. Bright field and fluorescent images showing isolated haustoria after 30 min incubation with WGA-FITC or ConA-Alexa 594. All images were collected on a Leica DMR epifluorescence microscope. (TIF) [file pone.0067150.s004.tif]
