# Supplementary material for: Strategies for Wheat Stripe Rust Pathogenicity Identified by Transcriptome Sequencing
Source: PLoS One. 2013 Jun 26;8(6):e67150. doi: 10.1371/journal.pone.0067150 (PMC3694141; doi:10.1371/journal.pone.0067150)
Supplement: Table S7 — Illumina sequencing data before and after mapping against the transcripts reference set. Three biological replicates were sequenced with Illumina for isolated haustoria (H) and germinated spores (S). The table shows the millions of reads obtained per replicate and the percentage of reads mapping against the transcripts reference set assembled from 454 haustoria and germinated spore data. (DOCX) [file pone.0067150.s011.docx]

| **Sample** | **Original reads (Mill)** | **After trimming (Mill)** | **Mapping in pairs (Mill)** | **Mapping in broken pairs (Mill)** | **% Mapping** | **Not mapping (Mill)** |
| --- | --- | --- | --- | --- | --- | --- |
| H1 | 38.5 | 37.5 | 20.4 | 1.8 | 60 | 14.3 |
| H2 | 70.3 | 68.3 | 41.7 | 4.2 | 67.2 | 22.3 |
| H3 | 53 | 51.9 | 30 | 2 | 61.6 | 18.7 |
| S1 | 110.2 | 108.1 | 73.7 | 7.1 | 74.7 | 25.3 |
| S2 | 120.5 | 118.1 | 79.6 | 8 | 74.1 | 28.1 |
| S3 | 115.6 | 113.4 | 77.9 | 7.4 | 75.2 | 25.9 |

**Table S7.**
