# Supplementary material for: Strategies for Wheat Stripe Rust Pathogenicity Identified by Transcriptome Sequencing
Source: PLoS One. 2013 Jun 26;8(6):e67150. doi: 10.1371/journal.pone.0067150 (PMC3694141; doi:10.1371/journal.pone.0067150)
Supplement: Text S1 — Supplementary text. (DOCX) [file pone.0067150.s017.docx]

**Text S1**

**Haustoria isolation by ConA chromatography column and Percoll gradients**

Haustoria were first isolated by affinity chromatography as in ([Hahn & Mendgen 1992](#_ENREF_3)). The preparations were analysed extensively by microscope to determine yield and purity. Light microscopy showed a mixture of single-cell haustoria, and contaminating entire and broken chloroplasts (Figure S4A). Chloroplast removal was not considered necessary because they yield an insignificant amount of RNA ([Catanzariti et al 2006](#_ENREF_2); [Hahn & Mendgen 1997](#_ENREF_4)) that in any case is not polyadenylated. Hemocytometer counts revealed that on average, 6.0 x 10^5^ haustoria were isolated per 15 g of heavily infected tissue, and that the haustoria:chloroplast ratio was approximately 1:15. Similar numbers have been reported from other rust fungi ([Catanzariti et al 2006](#_ENREF_2); [Hahn & Mendgen 1992](#_ENREF_3)) ConA fused to the fluorophore Alexa-fluor 594, and the lectin wheat germ agglutinin (WGA) fused to the fluorophore fluorescein isothiocyanate (FITC), were used to assess the purity and integrity of isolated haustoria (Figure S4B). Fluorescent images suggest that haustoria were isolated intact as single-cell structures, while bright field images showed that most of the haustoria retained their cytoplasmic contents, making them amenable to experimentation.

A second isolation technique employing a Percoll gradient step followed by cell sorting, was developed in parallel to obtain ultrapure isolated haustoria (Garnica and Rathjen, submitted). A Percoll gradient of whatever percent remarkably improved the haustorial yield, and the only contaminants detected after this step were chloroplasts. This protocol was used to isolate haustoria for Illumina data. More than 98% of sorted haustoria remained viable after the isolation process.

**454-pyrosequencing and assembly of germinated spores and haustoria transcriptomes**

Stripe rust isolated haustoria and germinated uredinospores (spores) transcriptomes were sequenced by single read pyrosequencing on a 454 GS-FLX titanium platform. A total of 301 Mb and 191 Mb of sequence data were generated in the form of 729,036 and 457,071 reads averaging 413 bp and 420 bp in length for haustoria and spores respectively. Prior to assembling all sequences, primers and sample specific tags sequences were removed from the dataset. In order to remove wheat contamination from the haustoria data set the Pst-130 draft genome ([Cantu et al 2011](#_ENREF_1)), the Pst-104E137A- draft genome generated by our group (Garnica, Rathjen, Studholme, unpublished results) and the *de novo* assembled spore transcriptome generated in this study were used as references to map haustoria reads. To assemble the *de novo s*pore reads, CLC genomics (CLC Bio 3.9) was used with optimized parameters: similarity 0.97 and overlap 0.5, producing 7,886 contigs. The number of reads per contig ranged between two and 27,778, and the level of coverage ranged from one to 23,719 (Figure S1, S2 and S3).

The non-mapping haustoria reads were assembled *de novo* and the resulting contigs screened by BLAST searches against the NCBI nucleotide and protein databases. BLAST results were curated manually and contigs showing hits to plant genes were removed, while the remaining contigs were retained as novel transcripts not included in the draft genome assemblies. The filtered haustorial reads were then assembled *de novo* using CLC genomics with the same parameters used to assemble spores transcripts, resulting in 12,846 contigs representing the haustorial transcriptome. The number of reads per contig ranged between two and 26,493 and the level of coverage ranged from one to 7,516. (Figures S1, S2 and S3).

Assembled contigs ranged between 200 bp and 6,854 bp for haustoria, and 200 bp and 4765 bp for spores, with average lengths of 776 bp and 794 bp respectively. Most of the contigs in both datasets (69.14% and 74.33%) are bigger than 500 bp, showing that 454 platform is useful for the generation of larger contigs, increasing the probability of predicting full-length open reading frames (ORFs).

Cantu D, Govindarajulu M, Kozik A, Wang M, Chen X, et al. 2011. Next generation sequencing provides rapid access to the genome of Puccinia striiformis f. sp. tritici, the causal agent of wheat stripe rust. *Plos One* 6:e24230

Catanzariti AM, Dodds PN, Lawrence GJ, Ayliffe MA, Ellis JG. 2006. Haustorially expressed secreted proteins from flax rust are highly enriched for avirulence elicitors. *Plant Cell* 18:243-56

Hahn M, Mendgen K. 1992. Isolation by Cona Binding of Haustoria from Different Rust Fungi and Comparison of Their Surface Qualities. *Protoplasma* 170:95-103

Hahn M, Mendgen K. 1997. Characterization of in planta induced rust genes isolated from a haustorium-specific cDNA library. *Mol Plant Microbe In* 10:427-37
